# Reflex Charging for Anion‐Derived Solid Electrolyte Interphase Formation

**DOI:** 10.1002/anie.4035251

**Published:** 2026-08-08

**Authors:** Gyeoul Seong, Min‐Gyun Kim, Eunyeong Jung, Yerin Kang, Hong‐I Kim, Kwon‐Hyung Lee, Kohei Shimokawa, Won Bo Lee, Sang‐Young Lee, Seung‐Hyeok Kim

**Affiliations:** ^1^ Department of Materials Science and Chemical Engineering Hanyang University Ansan Republic of Korea; ^2^ Department of Chemical and Biomolecular Engineering Yonsei University 50 Yonsei‐ro, Seodaemun‐gu, Seoul Republic of Korea; ^3^ School of Chemical and Biological Engineering Seoul National University 1 Gwanak‐ro, Seoul Republic of Korea; ^4^ Department of Engineering University of Cambridge Cambridge UK

**Keywords:** electric double layer, formation protocol, lithium metal battery, solid‐electrolyte interphase, solvation structure

## Abstract

Lithium (Li) metal offers exceptional energy density but suffers from unstable interphases that lead to dendrite growth and poor reversibility. Because the interphase is established during the earliest stages of operation, controlling its formation is critical for stabilizing Li metal electrodes. Here, we demonstrate that a reflex charging strategy, in which short discharge pulses are introduced during charging, dynamically reprograms the interfacial environment. This approach sustains anion enrichment near the electrode surface and shifts Li^+^ solvation from solvent‐dominated structures toward anion‐coordinated structures, thereby promoting the formation of an inorganic‐rich interphase. The resulting interphase reduces interfacial resistance and enables dense and uniform Li deposition. Consequently, the reflex charging‐based formation protocol exhibits markedly improved Li deposition/stripping reversibility, and greater resistance to degradation during cycling and calendar aging. In full cells paired with LiFePO_4_ (LFP) cathodes, the system retains 73% capacity after 500 cycles, demonstrating practical performance benefits. These findings establish formation protocol as an effective operation‐driven lever for controlling interfacial chemistry, offering a practical route that complements conventional materials‐based strategies for stabilizing Li metal batteries (LMBs).

## Graphical abstract

Reflex charging reprograms the electric double layer (EDL) during battery formation by periodically reversing electrode polarity, enabling persistent anion enrichment at the interface. This dynamic interfacial control shifts Li^+^ solvation toward anion‐coordinated states, promoting the formation of an inorganic‐rich, anion‐derived solid electrolyte interphase (SEI). As a result, Li deposition becomes more uniform and reversible.

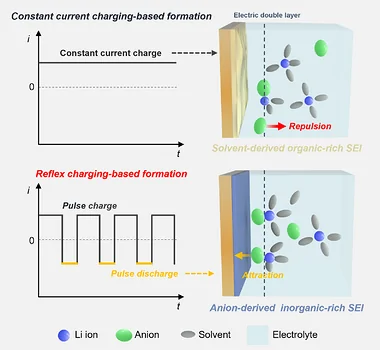

## 1 **|** Introduction

Lithium (Li) metal is widely regarded as an ideal anode for next‐generation batteries due to its high specific capacity (3860 mAh g^−1^) and low redox potential (−3.04 V vs. the standard hydrogen electrode) [1, 2]. However, its practical implementation remains limited by unstable interphases that lead to dendrite growth, parasitic reactions, and poor reversibility [3, 4, 5]. Because these interphases govern Li^+^ transport and mechanical/electrochemical stability, thereby crucially influencing dendrite formation and Li corrosion [6, 7, 8], their precise control is essential for enabling stable Li metal operation.

To address this challenge, extensive efforts have focused on electrolyte engineering and substrate engineering to promote the formation of stable interphases [9, 10, 11, 12]. These strategies have demonstrated significant progress, particularly in enabling inorganic‐rich solid electrolyte interphase (SEI) layers with high chemical/mechanical stability and low Li^+^ diffusion barriers [13, 14]. However, such materials‐based approaches often require complex optimization, remain system‐specific, and are difficult to generalize across different electrolyte chemistries and operating conditions.

Notably, the interphase is primarily formed during the earliest stages of battery operation, and its composition and structure are strongly influenced by the formation protocol [15, 16, 17]. Accordingly, the interphase formed during this process critically determines the electrochemical performance and long‐term stability of batteries. Various strategies, including current optimization and pulse charging, have been explored to control interphase formation [18, 19, 20]. Nevertheless, the formation process remains highly sensitive, making it challenging to precisely regulate the physicochemical characteristics of the interphase.

From a mechanistic perspective, interphase formation is governed not only by electrolyte and substrate properties but also by the structure of the electric double layer (EDL) at the electrode–electrolyte interface [21, 22]. The EDL differs fundamentally from the bulk electrolyte and dynamically evolves with the applied electrode potential [21, 23]. The specific adsorption of ionic species within this interfacial region dictates the preferential decomposition pathways of electrolyte components and, consequently, the resulting interphase chemistry. Therefore, controlling the structure and composition of the EDL is critical for constructing an ideal interphase that ensures stable Li deposition/stripping. Although the control of the EDL has largely relied on electrolyte and substrate engineering [24, 25, 26], these approaches are often indirect, costly, and difficult to generalize. Therefore, there remains a need for a universal and straightforward strategy to regulate interfacial structure and interphase formation.

In the pristine state of batteries, anions exhibit a strong propensity to accumulate near the anode surface [27]. However, under conventional charging conditions (i.e., constant current or constant voltage charging), the negatively polarized anode surface repels anions from the interfacial region, leading to an anion‐depleted environment [28, 29, 30]. This depletion suppresses anion reduction and instead promotes solvent‐dominated decomposition, resulting in the formation of organic‐rich SEI layers as schematically illustrated in Figure 1a.

**FIGURE 1:**
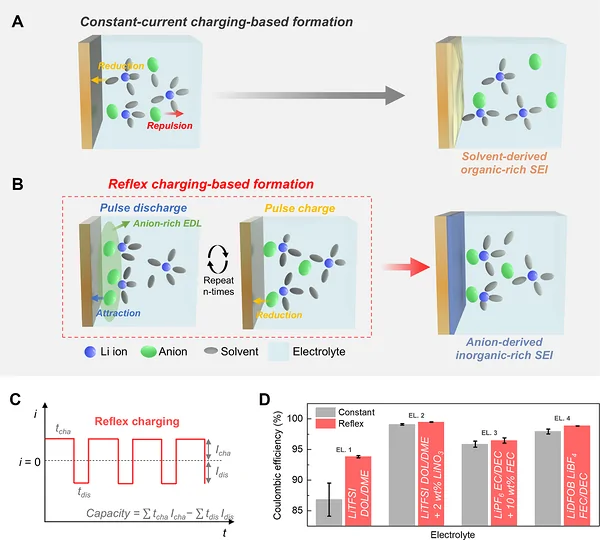
(a and b) Schematic illustration of the SEI formation mechanism in (a) CC charging‐based and (b) reflex charging‐based formation protocols. (c) Schematic illustration representing the process and variables of reflex charging. (d) Comparison of CE values evaluated by the Aurbach method under various electrolyte conditions after the CC charging‐based and reflex charging‐based formation protocols. Error bars represent the standard deviation from repeated Li||Cu measurements (*n* = 3), as summarized in Table S1.

Here, we propose that interfacial chemistry can be actively reprogrammed through electrochemical operation pathways. Specifically, we introduce a reflex charging‐based formation protocol, in which short discharge pulses are periodically applied during charging. This dynamic modulation of electrode polarity enables anion enrichment near the anode surface, fundamentally altering the interfacial solvation environment. As a result, Li^+^ coordination shifts toward anion‐involved structures, promoting the formation of inorganic‐rich SEI layers (Figure 1b). Theoretical investigations reveal that anions are expelled from the EDL region during conventional constant current (CC) charging. In contrast, applying short discharge pulses effectively establishes the anion‐enriched EDL. Additionally, reflex charging encourages the prevalence of contact ion pair (CIP) and aggregate (AGG)‐rich Li^+^ solvation structures within the EDL. As a consequence of this interfacial regulation, the reflex charging protocol induces the formation of LiF‐ and Li_2_O‐rich SEI layers with enhanced mechanical robustness. This interfacial stabilization enables dense and uniform Li deposition, suppresses corrosion, and improves calendar aging stability. Furthermore, in Li metal full cells paired with LiFePO_4_ (LFP) cathodes, the reflex‐charged Li anode delivers improved cycling stability, retaining 73% of its capacity after 500 cycles. This work establishes formation protocol as an effective operation‐driven lever for interphase engineering, demonstrating that interfacial chemistry can be governed not only by materials design but also by electrochemical operation. This perspective opens new opportunities to develop scalable operation‐driven strategies for stabilizing Li metal batteries (LMBs) and potentially other electrochemical systems.

## 2 **|** Results and Discussion

### 2.1 **|** Reflex Charging‐Based Formation Protocol for Stable Li Metal Anodes

Reflex charging is designed by introducing periodic short discharge pulses during the charging process, enabling dynamic modulation of electrode polarity. The key parameters include the charge and discharge durations (*t_cha_* and *t_dis_*), current densities (*I_cha_* and *I_dis_*), and the net charging capacity (*t_cha_ I_cha_* – *t_dis_ I_dis_*) (Figure 1c). In this study, the reflex charging was applied during the formation step to form anion‐derived, inorganic‐rich SEI layers.

To evaluate its effectiveness, the Coulombic efficiency (CE) values were measured in Li||Cu cells in various electrolytes (Figures 1d and S1; Table S1), including both ether‐ and carbonate‐based electrolyte systems (1 M lithium bis(trifluoromethanesulfonyl)imide (LiTFSI) in 1,3‐dioxolane (DOL)/1,2‐dimethoxyethane (DME) = 1/1 (v/v) (EL. 1), 1 M LiTFSI in DOL/DME = 1/1 (v/v) with 2 wt% lithium nitrate (LiNO_3_) (EL. 2), 1 M lithium hexafluorophosphate (LiPF_6_) in ethylene carbonate (EC)/diethyl carbonate (DEC) = 1/1 (v/v) with 10 wt% fluoroethylene carbonate (FEC) (EL. 3), and 0.6 M lithium difluoroborate (LiDFOB) and 0.6 M lithium tetrafluoroborate (LiBF_4_) in FEC/DEC = 1/2 (v/v) (EL. 4)). Under conventional CC charging‐based formation, the CE values were measured at 86.81% (EL. 1), 99.10% (EL. 2), 95.88% (EL. 3), 97.93% (EL. 4). In contrast, the reflex charging‐based formation showed enhanced CE values to 93.82% (EL. 1), 99.48% (EL. 2), 96.47% (EL. 3), 98.86% (EL. 4). Notably, for all electrolyte systems investigated, the reflex charging protocol consistently delivered higher average CE values than the CC charging method. These results suggest that the reflex charging‐based formation protocol can enhance average CE across the examined ether‐ and carbonate‐based electrolyte chemistries, although the magnitude of improvement depends on the electrolyte system. Based on these results, subsequent experiments in this study were conducted using the carbonate‐based electrolyte (EL. 4), providing stable operation with high‐voltage cathodes [31]. To identify optimal operating conditions, the parameters of the reflex charging protocol (*t_cha_*, *t_dis_*, *I_cha_*, and *I_dis_*) were systematically varied (Table S2). The condition yielding the highest average CE was selected as the optimum (Table S2), indicating that interfacial behavior can be effectively tuned by the electrochemical operating parameters.

### 2.2 **|** Theoretical Calculation Showing Anion Enrichment in the EDL by Reflex Charging

To clarify the anion‐enrichment effect of reflex charging, the number densities of ions and solvents in the EDL region were calculated by molecular dynamics (MD) simulation. Figure 2a illustrates snapshots of the structural evolution of ions near the electrode surface throughout the reflex charging protocol. To analyze spatial ion distribution in the EDL region (within 12.0 Å from the surface) [32, 33], the center‐of‐mass number density profiles of ions and solvent species are presented as a function of distance from the electrode, normalized with respect to the bulk densities of the electrolyte species (Figures 2b–d and S2 and Table S3). As shown in Figure 2b, the first peak of Li^+^ ions is located at ∼2–3 Å and the second peak of Li^+^ ions is located at ∼8 Å from the electrode surface. These characteristic peak positions provide a basis for comparing how the ion distributions within the EDL evolve during charge and discharge. It should be noted that a fixed‐charge planar graphene electrode based on the constant charge method (CCM), which is widely used as a standard model for electrified electrode–electrolyte interfacial systems [34, 35], was employed in the present MD simulations. Although this model does not explicitly describe electrolyte reduction reactions or SEI formation on reactive Li metal surfaces, it is well suited for capturing polarity‐driven ion/solvent redistribution and the evolution of interfacial solvation structures within the EDL prior to electrochemical reduction.

**FIGURE 2:**
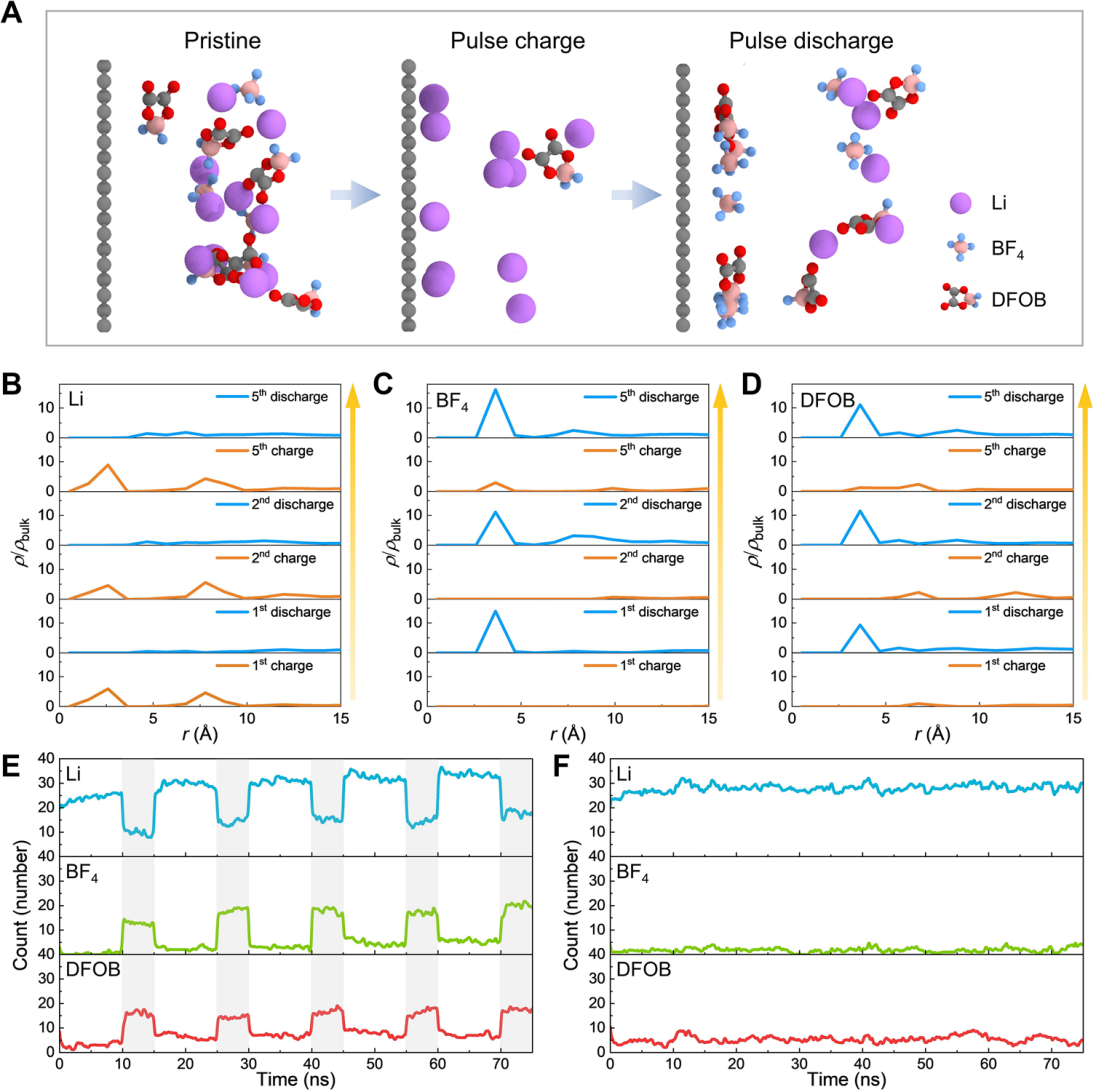
(a) MD simulation snapshots in EL. 4 at 363 K, showing the evolution of the EDL structure from neutral to negatively and positively charged surfaces under reflex charging. (b–d) Number density profiles of (b) Li^+^, (c) BF_4_^–^, and (d) DFOB^–^ ions during the first, second, and fifth cycles of the pulse charge–pulse discharge steps in the EDL region. (e and f) Time evolution of ion counts (Li^+^, BF_4_^−^, and DFOB^−^) in the EDL region under (e) reflex charging and (f) CC charging. In (e), the white and gray regions represent the pulse charge and pulse discharge steps, respectively.

During the first charge step, the negatively charged working electrode attracts Li^+^ cations to its surface, leading to a high concentration of cations near the electrode, whereas BF_4_^–^ and DFOB^–^ anions are repeled, resulting in their expulsion from the EDL region. These behaviors correspond to the first (∼2–3 Å) and second (∼8 Å) peaks in the density profiles for Li^+^ cations, while no distinct peak is observed for anions at this distance.

However, in the first discharge step, the polarity of the surface charges is reversed, making the working electrode positively charged. As a result, BF_4_^–^ and DFOB^–^ anions were attracted to the electrode surface, while Li^+^ cations were repelled from the EDL. A similar trend is observed during the subsequent pulse charge–pulse discharge steps, where ions carrying the same polarity as the electrode surface are repelled from the EDL. However, Figure 2c,d show that the first (∼2–3 Å) and second (∼8 Å) peaks of BF_4_^–^ and DFOB^–^ anions remain detectable even during the subsequent charge steps. This residual anion population becomes more pronounced as the cycle number increases. These results indicate that a fraction of anions is still retained within the EDL during charging, which can contribute to the formation of anion‐derived SEI layers.

The generality of this behavior was further confirmed in ether‐based electrolyte EL. 1 (Figures S3 and S4; Table S4), where similar trends of anion retention within the EDL were observed. These results demonstrate that reflex charging enables sustained anion enrichment at the interface, independent of electrolyte chemistry. Overall, these findings reveal that dynamic modulation of electrode polarity can reprogram the structure of the EDL, providing a direct and universal pathway to control interfacial ion distributions.

To further substantiate the anion‐enrichment effect induced by reflex charging, we quantitatively compared the number of ions within the EDL over an equivalent time window to that of CC charging (Figure 2e,f). Under reflex charging conditions, anions repeatedly re‐enter the EDL during each pulse discharge step, leading to a consistently higher anion population within the interfacial region over the entire charging period. In contrast, during conventional CC charging, the anion population within the EDL remains persistently low, indicating limited anion participation at the interface. These results clearly demonstrate that reflex charging dynamically reconfigures the EDL to maintain an anion‐enriched interfacial environment.

In addition, to more clearly verify the effect of reflex charging, we included a comparison with pulse charging by performing a pulse charge‐rest simulation under the same time ratio as the reflex charging protocol (Figures S5 and S6; Table S5). Compared with the rest step in the pulse charge‐rest simulation, the discharge step in the reflex protocol more effectively restores BF_4_^–^ and DFOB^–^ anions near the electrode surface by actively reversing the electrode polarity. The averaged ion count comparison further shows that the pulse charging protocol also partially increases the interfacial anion population compared with CC charging, but reflex charging maintains the highest average BF_4_^–^ and DFOB^–^ counts within the EDL region (Figure S7). This comparison highlights that polarity reversal, rather than simple relaxation during rest, is critical for regenerating an anion‐rich EDL during repeated cycling.

### 2.3 **|** Anion‐Coordinated Solvation Formation Induced by Reflex Charging

To further explore the effects of reflex charging on Li^+^ solvation shells, the solvation structures within the EDL were analyzed. The distributions of solvation states were quantified during pulse charge‐pulse discharge cycles (Figure 3a–d; Table S6). During the charge step of the first cycle, Li^+^ ions are predominantly coordinated by solvent molecules, corresponding to solvent‐separated ion pair (SSIP) (Figure 3a). In contrast, during the discharge step of the first cycle, SSIP species are absent in the EDL, while free anions and anion‐coordinated solvation species (CIP, AGG) dominate (Figure 3b). Notably, after repeating the pulse charge‐pulse discharge process five times, a substantial increase in the proportions of CIP and AGG species is observed even during the charging step, accompanied by a reduction in SSIP (Figure 3c). Furthermore, during the fifth discharge step, the relative fractions of CIP and AGG increase compared to that of free anions (Figure 3d).

**FIGURE 3:**
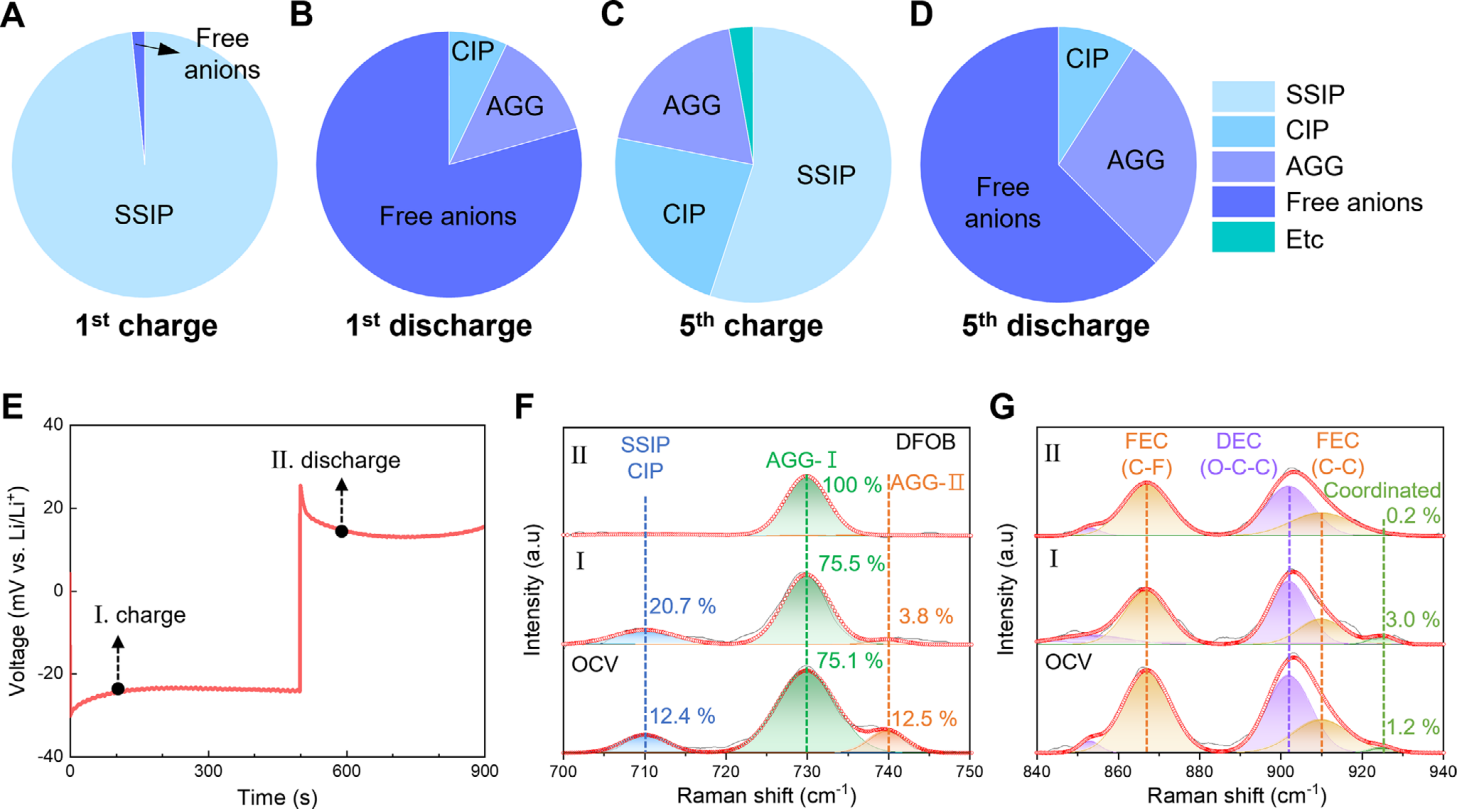
(a–d) Distribution of solvation structures in EL. 4 during (a) the charge step of the first cycle, (b) the discharge step of the first cycle, (c) the charge step of the fifth cycle, and (d) the discharge step of the fifth cycle, respectively. (e) Time–voltage plot of a Li||Cu cell for the in situ Raman test performed at a current density of 0.5 mA cm^−2^. (f and g) In situ Raman spectra of (f) DFOB^–^ anions and (g) FEC/DEC solvents, respectively, during Li deposition/stripping.

For comparison, solvation structures under CC charging were also examined under an equivalent charging duration (Figure S8 and Table S7). Li^+^ ions were predominantly present in SSIP (76.0%), with relatively low fractions of CIP (12.1%) and AGG (4.2%) species. In contrast, reflex charging significantly reduced the SSIP fraction (53.4%) while increasing the contributions of CIP (22.4%) and AGG (18.5%). These results demonstrate that reflex charging shifts interfacial solvation toward anion‐coordinated configurations, thereby promoting an anion‐rich interfacial environment. Importantly, this solvation‐structure change is accompanied by an increase in the absolute populations of BF_4_^–^ and DFOB^–^ anions within the EDL over an equivalent time window (Figure 2e,f). Therefore, the anion‐enriched interfacial solvation environment under reflex charging is supported by both the increased interfacial anion abundance and the enhanced anion participation in Li^+^ solvation.

Because the physical proximity of anions alone does not necessarily guarantee their preferential decomposition over solvents, density functional theory (DFT)‐based lowest unoccupied molecular orbital (LUMO) energy calculations were further performed to evaluate the reduction tendency of representative electrolyte species and Li^+^‐coordinated solvation structures derived from the MD‐predicted interfacial environments (Figure S9). In general, a lower LUMO energy indicates a higher tendency to accept electrons and undergo reduction [36]. The anion‐involved solvation structures exhibited lower LUMO energy levels than the solvent‐dominated counterparts, and their LUMO distributions were mainly localized on DFOB^–^. This DFOB^–^‐localized LUMO indicates that DFOB^–^ is the electronically favorable reduction site within the anion‐rich solvation structure. These results support that reflex charging not only enriches anions near the electrode surface but also promotes interfacial solvation structures with a higher tendency toward anion‐derived reductive decomposition, consistent with the experimentally observed inorganic‐rich SEI chemistry.

To capture the dynamic evolution of solvation structures, in situ Raman spectroscopy was performed during Li deposition/stripping. Figures 3e and S10 show the voltage variation of a Li||Cu cell during the charge–discharge process, and Figure 3f,g present the Raman spectra collected at the open‐circuit voltage (OCV) and specific points I, II. The Raman spectra of DFOB^–^ anions in the 700–750 cm^−1^ region reveal distinct spectral contributions assigned to combined SSIP/CIP and AGG solvation structures [37]. In this Raman analysis, SSIP and CIP are treated as a combined contribution. Accordingly, the Raman results are interpreted as evidence for the overall evolution of DFOB^–^‐involved solvation structures, mainly based on changes in the AGG‐related contribution, rather than as direct evidence for separate SSIP‐to‐CIP conversion. Under the OCV condition, the AGG‐related spectral contribution is dominant (87.6%), with a smaller combined SSIP/CIP contribution (12.4%). During the charge step I, the relative fraction of SSIP/CIP species (20.7%) increases, while the total AGG solvation structures (79.3%) are suppressed due to the attraction of Li^+^ ions and the repulsion of anions by the negatively charged working electrode surfaces. In contrast, during the discharge step II, the electrode becomes positively charged and strongly attracts anions, resulting in a dominant AGG‐related contribution.

This observation from the DFOB^–^ anion Raman spectra is further supported by the solvent Raman spectra (Figure 3g). Because the solvent‐coordinated contribution is very small and can be affected by uncertainty in peak deconvolution, the solvent Raman spectra are interpreted qualitatively rather than used for rigorous quantitative comparison. Within this limitation, the solvent Raman spectra show a decrease in the solvent‐coordinated contribution during the discharge step, which is consistent with the trend observed in the DFOB^–^ anion Raman spectra (Figure 3f).

### 2.4 **|** Anion‐Derived, Inorganic‐Rich SEI Layers Under Reflex Charging

To elucidate the impact of reflex charging on interphase properties, electrochemical impedance spectroscopy (EIS) combined with distribution of relaxation times (DRT) analysis was performed as a function of Li deposition capacity (Figure 4a–c). The impedance spectra reveal that the reflex charging exhibits consistently lower interfacial resistance compared to the CC charging. The DRT analysis further resolves the resistance contributions associated with the interphase and charge‐transfer processes. Two dominant features are observed, corresponding to SEI resistance (*R*_SEI_) and charge‐transfer resistance (*R*_ct_) [38, 39]. Notably, both contributions are significantly reduced under reflex charging.

**FIGURE 4:**
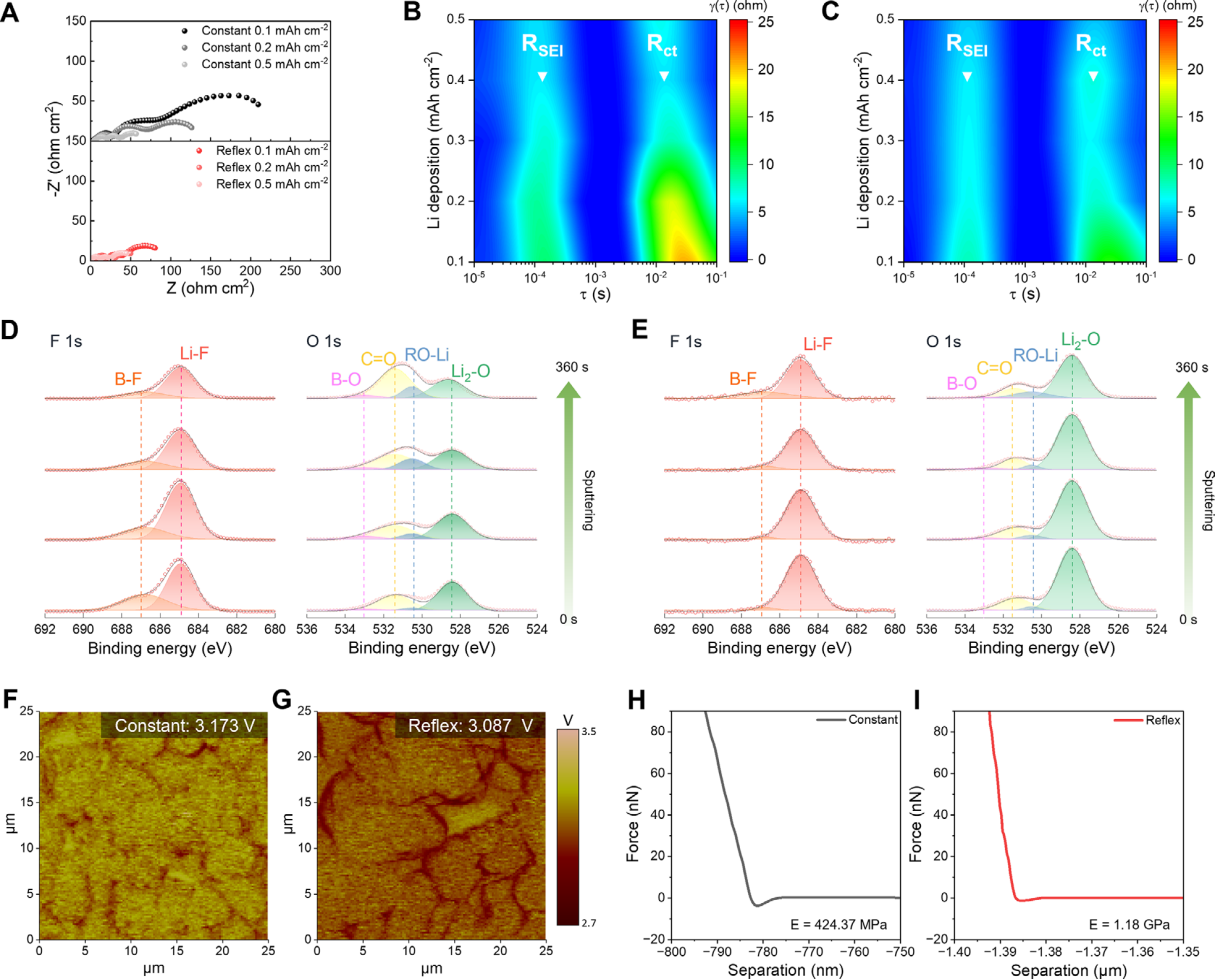
(a) Impedance spectra of Li||Li symmetric cells measured at 25°C as a function of Li deposition capacity under CC charging and reflex charging. (b and c) DRT analysis under (b) CC charging and (c) reflex charging. (d and e) XPS depth profiles of F 1s and O 1s after the Li deposition of 4 mAh cm^−2^ under (d) CC charging and (e) reflex charging. (f and g) KPFM surface potential maps and corresponding average surface potentials of Li deposits (areal capacity = 4 mAh cm^−2^) formed under (f) CC charging and (g) reflex charging. (h and i) F–D curves and the corresponding Young's modulus of Li deposits formed under (h) CC charging and (i) reflex charging.

X‐ray photoelectron spectroscopy (XPS) depth profiling was conducted after Li deposition (4 mAh cm^−2^) to probe the interphase composition (Figures 4d,e and S11). After the CC charging, the SEI shows high amounts of B–F (687.1 eV), B–O (533 eV), and RO–Li (530.5 eV) species. In contrast, the reflex charging leads to a substantial increase in inorganic species (Li–F (685 eV) and Li_2_–O (528.5 eV)) compared to the CC charging (Figure S12). Note that the abundant LiF and Li_2_O contents have been considered desirable for obtaining high reversibility and stable interfacial chemistry [40, 41]. Therefore, the enhanced LiF and Li_2_O contributions in the reflex‐charged SEI may be associated with the reduced *R*_SEI_ and *R*_ct_ observed in the EIS/DRT analysis. This interpretation is consistent with previous reports that inorganic‐rich interphases facilitate Li^+^ transport and lower interfacial resistance [42, 43]. Nevertheless, direct quantitative correlations among SEI composition, intrinsic ionic conductivity, and cycling stability require further systematic investigation.

To further examine the surface‐potential distribution and mechanical properties, atomic force microscopy (AFM) analyses were performed. Kelvin probe force microscopy (KPFM) measurements show that Li deposited under reflex charging exhibits a lower average surface potential than that formed under CC charging (3.087 vs. 3.173 V, Figure 4f,g). The surface‐potential distribution, pixel‐potential histograms and root‐mean‐square deviations were extracted from the KPFM maps (Figure S13). Therefore, the KPFM results are interpreted as evidence of a lower average surface‐potential state for the reflex‐charged Li. The lower surface potential suggests reduced interfacial reactivity associated with the formation of inorganic‐rich interphases [44, 45].

In addition, force–distance (F–D) curves show that Li deposits under reflex charging exhibit a steeper slope compared to the CC charging, corresponding to reduced deformation under load and thus higher mechanical rigidity (Figure 4h,i) [46]. Furthermore, Li deposits formed under reflex charging exhibit a higher Young's modulus (1.18 GPa) than those formed under CC charging (0.42 GPa). These results suggest that the formation of an inorganic‐rich SEI through reflex charging enhances the mechanical robustness of the Li surface.

### 2.5 **|** Electrochemical Performance of Li Metal Anodes Formed via Reflex Charging

Tafel analyses were performed in Li||Li symmetric cells after Li deposition (4 mAh cm^−2^) under CC charging and reflex charging‐based formation protocols (Figures 5a and S14). The exchange current density of the reflex‐charged cell (0.25 mA cm^−2^) is higher than that of the CC‐charged counterpart (0.16 mA cm^−2^). These results indicate that reflex charging‐based formation enhances the Li deposition/stripping kinetics, which can be attributed to the inorganic‑rich SEI layers [47].

**FIGURE 5:**
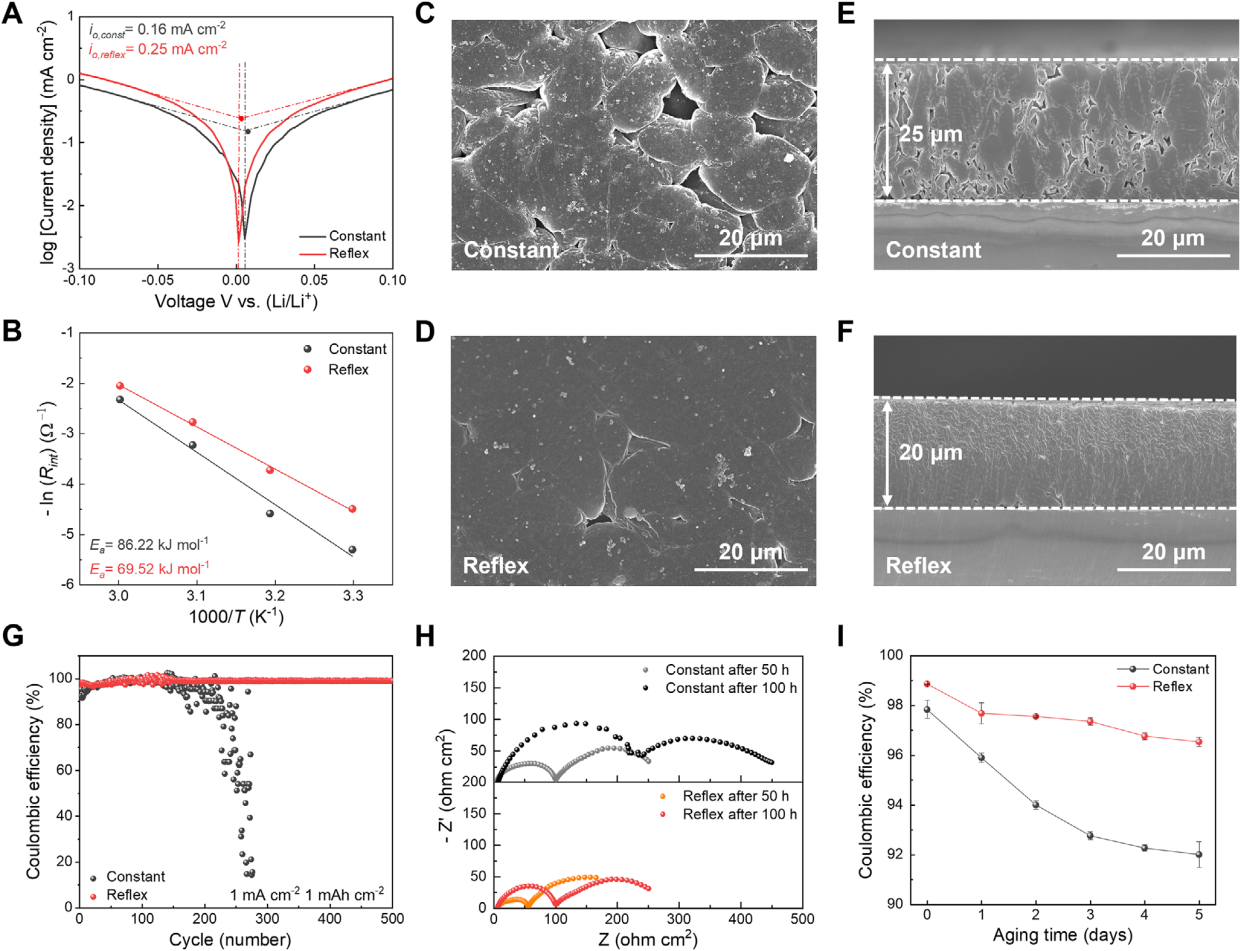
(a) Tafel plots and exchange current density of Li||Li symmetric cells under CC charging and reflex charging‐based formation protocols. (b) Arrhenius plots of the interfacial resistance measured in Li||Li symmetric cells from 30 to 60°C and the corresponding activation energies. (c–f) Surface and cross‐sectional SEM images of Li deposits under (c, e) CC charging and (d, f) reflex charging at a current density of 0.5 mA cm^−2^ to an areal capacity of 4 mAh cm^−2^. (g) CE of Li||Cu cells at a current density of 1 mA cm^−2^ with an areal capacity of 1 mAh cm^−2^. Prior to the CE measurements, CC charging and reflex charging‐based formation protocols were used. (h) Nyquist plots of Li||Li symmetric cells after 50 h and 100 h of symmetric cycling at 1 mA cm^−2^ with an areal capacity of 1 mAh cm^−2^. (i) CE of Li||Cu cells prepared for different aging times (0–5 days). Areal capacity of Li is 4 mAh cm^−2^ with a current density of 0.5 mA cm^−2^.

In addition, the Li^+^ transfer kinetics were further evaluated using temperature‐dependent resistance measurements based on the Arrhenius relationship (Figures 5b and S15). The activation energy (*E_a_*) associated with the interfacial Li^+^ transport is significantly lower under reflex charging (69.52 kJ mol^−1^) than under CC charging (86.22 kJ mol^−1^). This reduction in *E_a_* indicates that the reflex charging protocol effectively facilitates Li^+^ transfer kinetics by lowering the energy barrier for interfacial charge‐transfer and Li^+^ transport through the SEI.

Li deposition morphologies formed under CC charging and reflex charging protocols (areal capacity = 4 mAh cm^−2^) were analyzed using scanning electron microscopy (SEM) analysis (Figure 5c–f). Li deposited under CC charging exhibits a porous and heterogeneous morphology with a relatively thick layer (25 µm). In contrast, reflex charging yields dense and uniform Li deposits with a thickness of 20 µm, consistent with the theoretical value for an areal capacity of 4 mAh cm^−2^. Similar trends are observed across different electrolyte systems (EL. 1–EL. 3), showing the versatility of reflex charging‐based formation (Figure S16).

To investigate the electrochemical reversibility of Li deposition/stripping, CE values were evaluated in Li||Cu cells after CC charging and reflex charging‐based formation protocols (Figure 5g). The cells using CC charging‐based formation exhibit a pronounced decline in CE (94.2% after 200 cycles). In contrast, the cells using reflex charging‐based formation maintain high CE values (99.0%) after 500 cycles, indicating significantly improved cycling stability.

To assess corrosion resistance and long‐term interfacial stability, EIS was performed during symmetric cycling (Figure 5h). Although interfacial resistance increases over time for both systems, cells formed under reflex charging exhibit lower initial resistance and a slower increase during cycling compared to those formed under CC conditions, indicating suppressed interfacial degradation.

The impact of corrosion was further evaluated by CE measurements at different aging times (Figure 5i). Reflex‐charged Li yields a higher CE value compared to the CC‐charged Li (98.9% vs 97.8%), and the difference becomes more pronounced upon aging. While cells formed under CC conditions show a progressive decline in efficiency to 92.0% after 5 days, reflex‐charged cells retain higher efficiency (96.5%) under identical conditions. These results demonstrate that reflex charging effectively mitigates interfacial corrosion during aging, thereby enhancing long‐term stability and Li deposition/stripping reversibility. This behavior is consistent with the formation of stable inorganic‐rich interphases.

### 2.6 **|** Electrochemical Performance of Li Metal Full Cells

To evaluate the practical impact of reflex charging, Li deposited under this protocol was paired with an LFP cathode (areal capacity = 3.2 mAh cm^−2^) to construct Li metal full cells. The reflex‐charged cell exhibits a stable capacity retention (73% after 500 cycles), whereas the CC‐charged cell shows rapid degradation (41% after 300 cycles) (Figure 6a). To more clearly position the full cell performance of the reflex‐charged cell, these results were benchmarked against recent anion‐enrichment‐related Li metal full cell strategies (Table S8).

**FIGURE 6:**
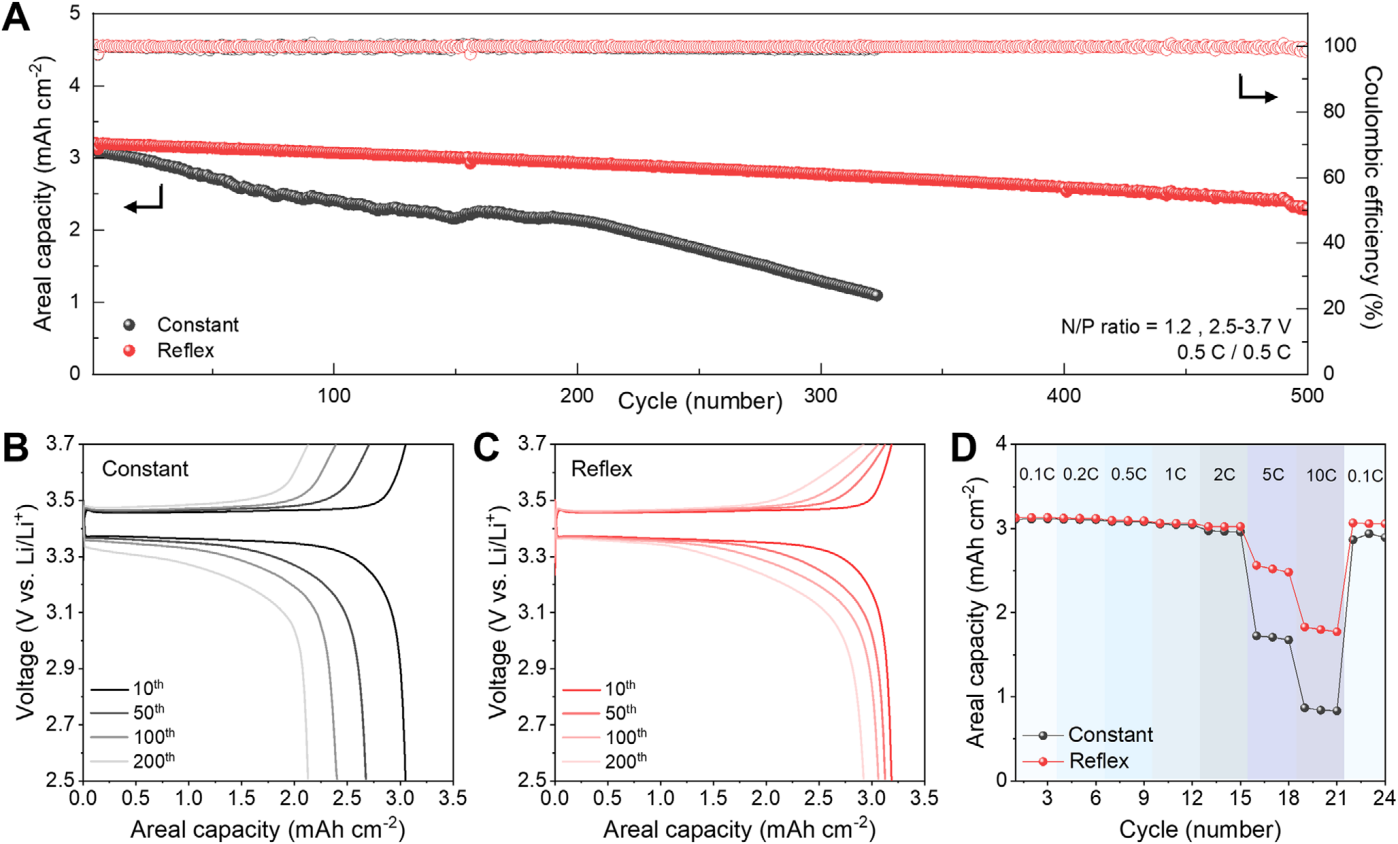
(a) Cycling performance of the Li metal full cells assembled with LFP cathodes (areal capacity = 3.2 mAh cm^−2^) and pre‐deposited Li metal anodes (areal capacity = 4 mAh cm^−2^) under either CC or reflex charging protocols. The cells were cycled at a charge/discharge current density of 0.5 C/0.5 C within a voltage window of 2.5–3.7 V. (b and c) Charge/discharge voltage profiles of the Li metal full cells as a function of cycle number with (b) CC charging and (c) reflex charging. (d) Rate capability of the Li metal full cell with CC charging and reflex charging protocols, in which the charge/discharge current densities varied from 0.1 to 10 C‐rate.

In addition to improved cycling stability, the reflex‐charged cell exhibits more stable voltage profiles during operation (Figure 6b,c), indicating reduced polarization. Post‐cycling impedance analysis further confirms enhanced interfacial stability (Figure S17). Consistently, surface morphology after cycling reveals that the reflex‐charged Li anode maintains a dense and uniform structure, whereas the CC counterpart shows a porous and degraded morphology (Figure S18).

To directly examine SEI evolution under full cell cycling conditions, the Li metal anodes were recovered from the Li||LFP full cells shown in Figure 6a after 20 cycles and analyzed by ex situ XPS depth profiling (Figure S19). Although both the CC‐ and reflex‐charged cells contain organic and inorganic species, the reflex‐charged cell still exhibits higher relative contributions of Li–F and Li_2_–O species in the F 1s and O 1s spectra, respectively. These results indicate that the anion‐derived, inorganic‐rich interphase induced by reflex charging is retained during repeated full cell cycling rather than being limited to the initial Li deposition process. Consistently, ex situ XPS depth profiling of anode‐free full cells, in which Li was supplied directly from the cathode, revealed the same enrichment of Li–F and Li_2_–O species under reflex charging, confirming that the SEI‐forming effect is preserved under full cell operating conditions (Figure S20).

The rate capability was further evaluated over a wide current range from 0.1 to 10 C (Figure 6d). The reflex‐charged cell delivers higher areal capacities than the CC cell under high‐rate conditions, particularly at 5 and 10 C. At 10 C, the reflex‐charged cell retains 56% of its capacity, whereas the CC counterpart retains only 23%.

To further evaluate the practical applicability of the reflex charging formation protocol, pouch‐type Li||Li[Ni_0.89_Co_0.05_Mn_0.05_Al_0.01_]O_2_ (NCMA) full cells were assembled under practical conditions. The cells employed an NCMA cathode with a theoretical areal capacity of 5.1 mAh cm^−2^, an N/P ratio of 1.3, and an E/C ratio of 5.0 g Ah^−1^, and were cycled at 0.5 C for both charge and discharge within a voltage range of 2.7–4.3 V. Under these conditions, the reflex‐charged pouch cell maintained an areal capacity of approximately 4.2 mAh cm^−2^ over 50 cycles with high CE, whereas the CC cell exhibited pronounced capacity decay and reached approximately 2.9 mAh cm^−2^ after 37 cycles (Figure S21).

Together with the interfacial analyses discussed above, these results support improved interfacial stabilization and Li deposition/stripping behavior under reflex charging‐based formation. Because reflex charging modifies the electrochemical formation protocol without introducing additional material components, it may provide a potentially scalable operation‐driven approach for stabilizing Li metal full cells.

## 3 **|** Conclusion

In summary, we demonstrate that reflex charging provides an effective formation strategy for engineering anion‐derived, inorganic‐rich SEI layers on Li metal anodes. By introducing short discharge pulses during charging, reflex charging promotes anion retention near the anode surface and shifts Li^+^ solvation within the EDL region toward anion‐coordinated configurations. This interfacial solvation regulation promotes the formation of an inorganic‐rich SEI with reduced interfacial resistance, enhanced Li^+^ transfer kinetics, and improved mechanical robustness. As a result, Li deposited under reflex charging exhibits dense and uniform morphology, improved reversibility, and enhanced resistance to interfacial degradation during aging and prolonged cycling. These interfacial advantages translate into markedly improved electrochemical performance, including stable capacity retention and enhanced high‐rate capability in Li metal full cells with practical LFP cathodes. More broadly, this study establishes the formation protocol as an effective and previously underexplored design parameter for controlling interfacial chemistry. By decoupling interphase engineering from materials design, reflex charging provides a potentially scalable operation‐driven strategy for stabilizing Li metal electrodes. At the same time, further optimization of the reflex charging protocol should consider not only the formation of inorganic‐rich SEI components but also the balance between inorganic and organic phases within the SEI. Identifying the optimal organic/inorganic balance in the SEI remains an important future direction for further improving the mechanical durability, ionic transport, and long‐term stability of Li metal interfaces. By demonstrating that interphase chemistry can be regulated through electrochemical operation rather than solely through materials modification, this work offers a foundation for developing operation‐driven strategies for stabilizing Li metal electrodes and potentially extending interfacial control to other electrochemical systems.

## Author Contributions

**Gyeoul Seong**: conceptualization, investigation, formal analysis, visualization, writing – original draft. **Min‐Gyun Kim**: conceptualization, investigation, data curation, formal analysis, writing – original draft. **Eunyeong Jung**: software, formal analysis, data curation, writing – review and editing. **Yerin Kang**: writing – review and editing. **Hong‐I Kim**: writing – review and editing. **Kwon‐Hyung Lee**: writing – review and editing. **Kohei Shimokawa**: writing – review and editing. **Won Bo Lee**: supervision, resources, writing – review and editing. **Sang‐Young Lee**: supervision, resources, writing – review and editing. **Seung‐Hyeok Kim**: conceptualization, supervision, project administration, funding acquisition, writing – review and editing.

## Conflicts of Interest

The authors declare no conflicts of interest.

## Supporting information




**Supporting File**: anie74097‐sup‐0001‐SuppMat.docx.

## Data Availability

The data that supports the findings of this study are available in the Supporting Information of this article.
