# Supplementary material for: Reflex Charging for Anion‐Derived Solid Electrolyte Interphase Formation
Source: Angew Chem Int Ed Engl. 2026 Aug 8;65(41):e4035251. doi: 10.1002/anie.4035251 (PMC13638108; doi:10.1002/anie.4035251)
Supplement: Supplementary file 1 [file ANIE-65-e4035251-s001.docx]

Supporting Information
©Wiley-VCH 2026
69451 Weinheim, Germany

**Reflex Charging for Anion-Derived Solid Electrolyte Interphase Formation**

Gyeoul Seong,^†[a]^ Min-Gyun Kim,^†[b]^ Eunyeong Jung,^†[c]^ Yerin Kang,^[a]^ Hong-I Kim,^[b]^ Kwon-Hyung Lee,^[d]^ Kohei Shimokawa,^[d]^ Won Bo Lee,^*[c]^ Sang-Young Lee,^*[b]^ and Seung-Hyeok Kim^*[a]^

^[a]^Department of Materials Science and Chemical Engineering, Hanyang University, Ansan 15588, Republic of Korea

^[b]^Department of Chemical and Biomolecular Engineering, Yonsei University, 50 Yonsei-ro, Seodaemun-gu, Seoul 03722, Republic of Korea

^[c]^School of Chemical and Biological Engineering, Seoul National University, 1 Gwanak-ro, Seoul 08826, Republic of Korea

^[d]^Department of Engineering, University of Cambridge, Cambridge, CB3 0FS, United Kingdom

*Correspondence and requests for materials should be addressed to wblee@snu.ac.kr (W. B. L), syleek@yonsei.ac.kr (S.-Y. L), and seunghyeok94@hanyang.ac.kr (S.-H. K).

^†^ These authors contributed equally to this work.

**Table of Contents**

1 Experimental Methods
2 Supporting Figures
3 Supporting Tables
4 References

**1. Experimental Methods**

**Materials and electrolytes preparation**

Fluoroethylene carbonate (FEC) (battery grade, ≥99%) and diethyl carbonate (DEC) (99%) were purchased from Sigma-Aldrich and dried over activated molecular sieves (4 Å, Sigma-Aldrich) before use. Lithium nitrate (LiNO_3_) (>99.99%), lithium difluoroborate (LiDFOB) (>99.99%) and lithium tetrafluoroborate (LiBF_4_) (>99.99%) salts were purchased from Sigma-Aldrich. 1 M lithium bis(trifluoromethanesulfonyl)imide (LiTFSI) in 1,3-dioxolane (DOL)/1,2-dimethoxyethane (DME) = 1/1 (v/v), 1 M lithium hexafluorophosphate (LiPF_6_) in ethylene carbonate (EC)/DEC = 1/1 (v/v) were obtained from Enchem (Korea). EL. 1 electrolytes were prepared by dissolving 1 M LiTFSI in DOL/DME = 1/1 (v/v). EL. 2 electrolytes were prepared by dissolving 2 wt% LiNO_3_ in 1 M LiTFSI in DOL/DME = 1/1 (v/v). EL. 3 electrolytes were prepared by dissolving 10 wt% FEC in 1 M LiPF_6_ in EC/DEC = 1/1 (v/v). EL. 4 electrolytes were prepared by dissolving 0.6 M LiDFOB and 0.6 M LiBF_4_ in FEC/DEC = 1/2 (v/v). All cells were fabricated in an argon-filled glovebox (O_2_ and H_2_O < 0.1 ppm).

**Preparation of the electrodes**

The electrode slurries were prepared with a composition ratio of LiFePO_4_ (LFP) (Ubatt)/carbon black additive (Super C65)/polyvinylidene fluoride (PVDF, Solvay) = 90/5/5 (w/w/w), using N-methyl-2-pyrrolidone (NMP, Aldrich) as the solvent. The electrodes were fabricated by casting the electrode slurry on an Al current collector. The cast electrode slurry was dried at 120 °C for 1 h, followed by roll-pressing at 60 °C. The electrodes were vacuum-dried at 120 °C for 12 h before the cell assembly.

**Characterizations**

The surface and cross-sectional morphologies of Li metal were characterized using field-emission scanning electron microscopy (SEM) (S-4800, Hitachi). The *in-situ* Raman spectrum was obtained using a Raman spectrometer (LabRam Aramis, Horiba) with a laser excitation wavelength of 785 nm and power of approximately 2 mW. Voltage profile data of the *in-situ* Raman cells were recorded using a potentiostat/galvanostat (VSP classic, Bio-Logic). To minimize the influence of the bulk electrolyte, measurements were conducted with a focus on the electrode–electrolyte interfacial region. The composition of the solid electrolyte interphase (SEI) layer in the X-ray photoelectron spectroscopy (XPS) profile was confirmed using XPS (K-alpha, Thermo U. K.). Atomic force microscopy (AFM) measurements were conducted using an NX10 system (Park Systems, Korea) inside a glovebox. Kelvin probe force microscopy (KPFM) mode was employed to map the relative surface potential of dried Li deposits over a scan area of 25 × 25 μm^2^ at a scan rate of 0.15 Hz with a resolution of 128 × 128 pixels, using a Multi75E-G cantilever. In addition, force–distance (F–D) spectroscopy was performed to evaluate the mechanical properties of the SEI using an FMR cantilever. Young’s modulus was calculated from the approach curves of the F–D measurements using the Hertz contact model implemented in the XEI software. The fitting range was selected from the approach curve between the maximum force and approximately 20% of the maximum force relative to the non-contact baseline, where the baseline corresponds to the zero-force region before tip–sample contact. A Poisson’s ratio of 0.38, close to the reported value for Li metal,^[1]^ was used as an approximation for the SEI formed on Li deposits. The tip curvature radius used for fitting was set to 10 nm based on the FMR cantilever specification (<10 nm).

**Electrochemical measurements**

Li metal was first deposited in Li||Cu cells at a current density of 0.5 mA cm^–2^ under either constant current (CC) charging or reflex charging protocols. Unless otherwise specified, Li deposition was carried out to an areal capacity of 4 mAh cm^–2^. For the electrochemical impedance spectroscopy (EIS)/distribution of relaxation times (DRT) analysis shown in Figure 4a–c, Li deposition was instead performed to varying areal capacities of 0.1, 0.2, and 0.5 mAh cm^–2^. Li deposition was performed using a Li (200 μm, Honjo Chemical Co.)||Cu cell with a liquid electrolyte amount of 70 μL. The Coulombic efficiency (CE) of the current collectors was measured using a Li||Cu cell. The EIS data of the cells were recorded using a potentiostat/galvanostat (VMP-300, Bio-Logic) in the frequency range of 10^–2^ to 10^6^ Hz at an applied voltage of 10 mV. Li metal full cells were fabricated by coupling pre-deposited Li on Cu (areal capacity = 4 mAh cm^–2^) with the LFP cathode (active material loading = 21.6 mg cm^–2^; areal capacity = 3.2 mAh cm^–2^) to obtain a negative-to-positive (N/P) ratio of 1.2. The electrochemical performance of the cells was investigated using 2032-type coin cells and measured at ambient temperature under various charge/discharge current densities and a voltage range of 2.5–3.7 V for LFP, using a cycle tester (PNE Solution). All cells were fabricated in an argon-filled glovebox (O_2_ and H_2_O < 0.1 ppm) and one layer of Celgard 2325 separator was used. The activation energy for Li deposition was calculated using the Arrhenius relationship:

$$\frac{1}{R_{int}}\propto Aexp(-\frac{E_{a}}{RT})$$

Here, *E_a_* is the activation energy, *T* is the absolute temperature, *R* is the gas constant, *R_int_* denotes the interfacial Li^+^ transfer resistance, and *A* is the pre-exponential factor.^[2, 3]^

**Molecular dynamics simulation details**

Molecular dynamics (MD) simulations were performed using the GROMACS software package version 2020.6 for the LiBF_4_/LiDFOB in DEC/FEC-based electrolyte system.^[4]^ The Optimized Potentials for Liquid Simulations all-atom (OPLS-AA) force field,^[5]^ generated by the LigParGen server was used for the solvents,^[6]^ while the Canongia Lopes & Pádua (CL&P) force field was applied for the Li^+^ and BF_4_^–^ ions.^[7]^ Parameters for DFOB⁻ were derived from Fischer et al.,^[8]^ and bonded parameters were adopted from Wang et al.^[9]^ Partial charges of the ions were uniformly scaled by a factor of 0.8 to account for electrostatic polarization and screening in non-polarizable force fields.^[10, 11]^ Snapshots from the MD simulations were visualized using OVITO.^[12]^

The initial configuration was constructed in a box with dimensions of $4.2\times4.2\times10.8 nm^{3}$, containing 66 LiBF_4_ and 66 LiDFOB ion pairs and 500 DEC and 500 FEC solvent molecules, located between two fixed, parallel graphene electrodes. The graphene sheets were generated using Visual Molecular Dynamics (VMD) software and consisted of 680 carbon atoms.^[13]^ To equilibrate the electrolyte density under desired pressure and temperature conditions, an NPT simulation was conducted at 1 bar using the Berendsen barostat,^[14]^ followed by an NVT simulation at 363 K through a velocity-rescaling thermostat both with a time constant of 2 fs.^[15]^ To minimize periodic image interactions, a vacuum layer was introduced by expanding the simulation box by 200% along the z-direction, while applying periodic boundary conditions in all directions.^[16]^

The reflex charging protocol was applied to the simulated interfacial system over five cycles. Each cycle began with a 0.5 ns equilibration step with neutral electrodes. This was followed by a 10 ns charge step, applying a surface charge density of ±1.08 e/nm^2^,^[16, 17]^ with charges uniformly distributed across the carbon atoms using the constant charge method (CCM).^[18]^ After another 0.5 ns equilibration step, a 5 ns discharge step was performed, with the electrode surface charges reversed. This equilibration-charge-equilibration-discharge cycle was repeated five times under NVT conditions, with the duration of the charging step being twice that of the discharging step, to simulate experimental conditions.

Throughout the cycles, energy minimization was conducted using the steepest descent method with a convergence tolerance of 1000.0 kJ/mol/nm. The velocity-rescaling thermostat maintained the system temperature at 363 K,^[15]^ with an integration timestep of 2 fs. Bonds involving hydrogen atoms were constrained using the SHAKE and LINCS algorithms.^[19, 20]^ A Verlet cutoff scheme was applied to generate pair lists, with a cutoff distance of 1.2 nm for both nonbonded Lennard-Jones and Coulombic interactions. Long-range electrostatic interactions were computed using the particle mesh Ewald (PME) method,^[21]^ with a real-space cutoff of 1.2 nm and a Fourier grid spacing of 0.12 nm. All graphene layers were kept fixed (frozen) throughout the simulations.

The fixed graphene electrodes used in the CCM framework represent idealized electrified surfaces rather than chemically reactive Li metal surfaces. Therefore, the present MD simulations were designed to isolate the electrostatic redistribution of ions and solvent molecules in the electric double layer (EDL) under electrode polarization and polarity reversal, rather than to directly simulate electrolyte reduction, Li nucleation, or SEI formation.

**Density functional theory calculation details**

Geometry optimizations and subsequent harmonic frequency calculations were performed at the ωB97X-D3/def2-TZVP level,^[22, 23]^ followed by single-point energy calculations at the ωB97X-D3/def2-TZVPD level using ORCA 5.0.1.^[22–26]^ The DefGrid3 integration grid and tight self-consistent field (SCF) convergence criteria were used throughout all density functional theory (DFT) calculations. Solvent effects were treated implicitly using the conductor-like polarizable continuum model (CPCM),^[27, 28]^ with a dielectric constant of ε = 78.4, corresponding to FEC,^[29]^ the higher dielectric component of the binary solvent mixture.

**2. Supporting Figures**

**Figure S1.** (a–d) CE profiles of Li deposition measured in Li||Cu cells under CC charging and reflex charging protocols in (a) EL. 1, (b) EL. 2, (c) EL. 3, and (d) EL. 4, corresponding to Figure 1d.

**Figure S2.** (a, b) Number density profiles of (a) DEC and (b) FEC solvent molecules during the 1^st^, 2^nd^, and 5^th^ charge–discharge cycles in the EDL of EL. 4.

**Figure S3.** MD simulation snapshots of the EDL in EL. 1 during the reflex charging protocol, showing the structural evolution of interfacial ionic configurations during repeated charge–discharge steps.

**Figure S4.** (a–d) Number density profiles of (a) Li^+^, (b) TFSI^–^, (c) DOL, and (d) DME species in the EDL during the 1^st^, 2^nd^, and 5^th^ charge–discharge cycles in EL. 1.

**Figure S5.** (a–e) Number density profiles of (a) Li^+^, (b) BF_4_^–^, and (c) DFOB^–^ ions, as well as (d) DEC and (e) FEC solvent molecules during the 1^st^, 2^nd^, and 5^th^ pulse charge–rest cycles in the EDL region of EL. 4 under the pulse charging protocol.

**Figure S6.** Time evolution of ion counts (Li^+^, BF_4_^–^, and DFOB^–^) in the EDL region under the pulse charge–rest protocol. The white and gray regions represent the pulse charge and rest steps, respectively.

**Figure S7.** Average ion counts of Li^+^, BF_4_^–^, and DFOB^–^ in the EDL region under CC charging, pulse charging and reflex charging protocols.

**Figure S8.** (a, b) Distributions of solvation structures in the EDL of EL. 4 under CC charging, showing the distributions (a) before CC charging and (b) after CC charging for a total charging duration equivalent to that of 5 charge–discharge cycles under the reflex charging protocol.

**Figure S9**. (a, b) DFT-calculated lowest unoccupied molecular orbital (LUMO) energy levels and orbital distributions of (a) individual electrolyte species and (b) Li^+^-coordinated species and representative solvation structures derived from the MD-predicted EDL environments. The lower LUMO energy and DFOB^–^-localized LUMO distribution in the anion-rich solvation structure indicate its higher tendency toward anion-derived reductive decomposition.

**Figure S10.** Full Raman spectra of EL. 4 during the charge–discharge cycling of Li plating/stripping. The spectra provide the full spectral range corresponding to the selected regions analyzed in Figure 3f, g.

**Figure S11.** (a, b) XPS depth profiles obtained after Li deposition of 4 mAh cm^–2^ via (a) CC charging and (b) reflex charging.

**Figure S12.** Relative peak-area ratios of LiF and Li_2_O components obtained from the fitted F 1s and O 1s XPS spectra, respectively, after Li deposition of 4 mAh cm^–2^ under CC charging and reflex charging protocols. The values represent the relative contribution of LiF within the fitted F 1s spectrum and Li_2_O within the fitted O 1s spectrum at each sputtering time.

**Figure S13.** Pixel-potential histograms of Li metal deposits formed under CC charging and reflex charging, extracted from the KPFM maps shown in Figure 4f,g. The root-mean-square deviations of the surface potential were 0.051 V for the CC-charged Li and 0.054 V for the reflex-charged Li, indicating comparable potential dispersion within the measured regions.

**Figure S14.** Cyclic voltammetry curves of Li||Li symmetric cells containing electro-deposited Li prepared under CC charging and reflex charging protocols.

**Figure S15.** (a, b) Temperature-dependent impedance spectra of Li||Li symmetric cells containing electro-deposited Li prepared under (a) CC charging and (b) reflex charging.

**Figure S16.** (a–f) SEM images of Li metal deposited via different charging protocols in (a, b) EL. 1, (c, d) EL. 2, and (e, f) EL. 3 after Li deposition at a current density of 0.5 mA cm^–2^ to an areal capacity of 4 mAh cm^–2^.

**Figure S17.** Nyquist plots of Li metal full cells assembled with Li metal anodes pre-deposited via either CC charging or reflex charging protocols, measured after 100 cycles at 0.5 C.

**Figure S18.** (a, b) Surface SEM images of Li metal anodes deposited via (a) CC charging and (b) reflex charging after 100 cycles in Li metal full cells.

**Figure S19.** (a, b) XPS depth profiles of SEI layers on cycled Li metal anodes in Li||LFP full cells after 20 cycles under (a) CC charging and (b) reflex charging.

**Figure S20.** (a, b) XPS depth profiles of F 1s, O 1s and C 1s in anode-free full cells (Cu||LFP) after charging using (a) CC charging and (b) reflex charging.

**Figure S21.** Cycling performance of Li||Li[Ni_0.89_Co_0.05_Mn_0.05_Al_0.01_]O_2_ (NCMA) pouch cells under practical conditions with a cathode areal capacity of 5.1 mAh cm^–2^, an N/P ratio of 1.3, and an electrolyte-to-capacity (E/C) ratio of 5.0 g Ah^–1^.

**3. Supporting Tables**

**Table S1.** CE values measured by the Aurbach method in Li||Cu cells under CC charging and reflex charging protocols in different electrolyte systems (EL. 1–EL. 4), corresponding to Figure 1d.

| **Electrolyte** | **Formation protocol** | **Coulombic efficiency by Aurbach method (%)** | | | **Average (%)** |
| --- | --- | --- | --- | --- | --- |
|  |  | **1^st^** | **2^nd^** | **3^rd^** |  |
| **EL. 1)** 1 M LiTFSI DOL/DME | Constant current | 82.50 | 88.41 | 89.51 | 86.81 |
|  | Reflex | 93.65 | 93.82 | 93.99 | 93.82 |
| **EL. 2)** 1 M LiTFSI DOL/DME + 2 wt% LiNO_3_ | Constant current | 99.06 | 99.02 | 99.23 | 99.10 |
|  | Reflex | 99.51 | 99.47 | 99.47 | 99.48 |
| **EL. 3)** 1 M LiPF_6_ EC/DEC + 10 wt% FEC | Constant current | 95.43 | 96.37 | 95.84 | 95.88 |
|  | Reflex | 96.91 | 95.95 | 96.55 | 96.47 |
| **EL. 4)** 0.6 M LiDFOB 0.6 M LiBF_4_ FEC/DEC | Constant current | 97.31 | 98.33 | 98.15 | 97.93 |
|  | Reflex | 98.85 | 98.88 | 98.86 | 98.86 |

**Table S2.** CE values measured by the Aurbach method in EL. 4 while varying the current densities and times of the charge and discharge steps in the reflex charging protocol.

| **Charging current, *I*_cha_**  **(mA cm^–2^)** | **Charging time, *t*_cha_**  **(s)** | **Discharging current, *I_dis_***  **(mA cm^–2^)** | **Discharging time, *t_dis_***  **(s)** | **Coulombic efficiency by Aurbach method (%)** | | | **Average (%)** |
| --- | --- | --- | --- | --- | --- | --- | --- |
|  |  |  |  | **1^st^** | **2^nd^** | **3^rd^** |  |
| 0.1 | 10 | 0.1 | 5 | 98.29 | 98.64 | 98.80 | 98.58 |
| 0.1 | 10 | 0.5 | 1 | 98.58 | 98.80 | 98.22 | 98.53 |
| 0.1 | 10 | 1 | 0.5 | 98.66 | 98.34 | 98.70 | 98.57 |
| 0.1 | 60 | 0.1 | 30 | 98.89 | 97.85 | 98.84 | 98.53 |
| 0.1 | 60 | 0.5 | 6 | 98.60 | 98.42 | 98.77 | 98.60 |
| 0.1 | 60 | 1 | 3 | 98.60 | 98.49 | 98.51 | 98.53 |
| 0.5 | 10 | 0.1 | 25 | 98.79 | 98.64 | 98.49 | 98.64 |
| 0.5 | 10 | 0.5 | 5 | 98.85 | 98.88 | 98.86 | 98.86 |
| 0.5 | 10 | 1 | 2.5 | 98.90 | 98.94 | 98.70 | 98.85 |
| 0.5 | 60 | 0.1 | 150 | 97.92 | 97.99 | 98.20 | 98.04 |
| 0.5 | 60 | 0.5 | 30 | 97.97 | 98.29 | 99.06 | 98.44 |
| 0.5 | 60 | 1 | 15 | 97.62 | 98.65 | 98.39 | 98.22 |
| 1 | 10 | 0.1 | 50 | 98.20 | 97.95 | 98.05 | 98.07 |
| 1 | 10 | 0.5 | 10 | 98.20 | 98.35 | 98.70 | 98.42 |
| 1 | 10 | 1 | 5 | 97.98 | 97.92 | 98.54 | 98.15 |
| 1 | 60 | 0.1 | 300 | 98.01 | 97.89 | 97.90 | 97.93 |
| 1 | 60 | 0.5 | 60 | 98.07 | 98.55 | 98.17 | 98.26 |
| 1 | 60 | 1 | 30 | 98.01 | 98.35 | 97.72 | 98.03 |

**Table S3.** Averaged number density values of Li^+^, BF_4_^–^, DFOB^–^, DEC, and FEC species within the EDL during the 1^st^, 2^nd^, and 5^th^ charge–discharge cycles in EL. 4.

**Li^+^ ρ/ρ_bulk_**

| **r(Å)** | **1^st^ charge** | **1^st^ discharge** | **2^nd^ charge** | **2^nd^ discharge** | **5^th^ charge** | **5^th^ discharge** |
| --- | --- | --- | --- | --- | --- | --- |
| 0.51881 | 0 | 0 | 0 | 0 | 0 | 0 |
| 1.55644 | 2.311818 | 0 | 2.196818 | 0 | 2.688409 | 0 |
| 2.59406 | 5.925227 | 0 | 4.6075 | 0 | 8.908712 | 0 |
| 3.63168 | 0.001515 | 0.021671 | 0.000833 | 0.090776 | 0.005227 | 0.111083 |
| 4.66931 | 0.011591 | 0.469033 | 0.091439 | 1.145987 | 0.103409 | 1.453776 |
| 5.70693 | 0.217955 | 0.280056 | 0.483864 | 0.437209 | 0.446591 | 0.987622 |
| 6.74455 | 0.907348 | 0.564052 | 0.828788 | 0.910182 | 0.948333 | 1.831427 |
| 7.78218 | 4.618409 | 0.142301 | 5.609242 | 0.806373 | 4.317121 | 0.845624 |
| 8.8198 | 1.636061 | 0.391139 | 2.418712 | 1.0934 | 2.609318 | 1.121133 |
| 9.85743 | 0.164015 | 0.444634 | 0.294318 | 1.1669 | 0.419924 | 1.104009 |
| 10.89505 | 0.342879 | 0.816375 | 0.680682 | 1.468324 | 0.601667 | 1.302533 |
| 11.93267 | 0.628712 | 1.09234 | 1.614394 | 1.196451 | 1.155227 | 1.374517 |
| 12.9703 | 0.425 | 0.821983 | 1.31947 | 0.82759 | 1.040758 | 1.155534 |
| 14.00792 | 0.325682 | 0.784248 | 0.869621 | 0.617851 | 0.890227 | 0.966557 |
| 15.04554 | 0.432955 | 1.063394 | 0.922652 | 0.70211 | 0.973485 | 0.847594 |
| 16.08317 | 0.688333 | 1.209181 | 0.855 | 0.709081 | 0.921818 | 0.835925 |
| 17.12079 | 0.95197 | 1.270406 | 0.967045 | 0.534955 | 0.801591 | 1.075367 |
| 18.15842 | 0.97697 | 1.258737 | 1.066894 | 0.623458 | 0.782955 | 1.201452 |
| 19.19604 | 1.016061 | 1.077337 | 0.849318 | 0.782732 | 0.824015 | 1.223881 |
| 20.23366 | 1.058939 | 1.011566 | 0.696136 | 0.871841 | 0.827727 | 1.255857 |

**BF_4_^–^ ρ/ρ_bulk_**

| **r(Å)** | **1^st^ charge** | **1^st^ discharge** | **2^nd^ charge** | **2^nd^ discharge** | **5^th^ charge** | **5^th^ discharge** |
| --- | --- | --- | --- | --- | --- | --- |
| 0.51881 | 0 | 0 | 0 | 0 | 0 | 0 |
| 1.55644 | 0 | 0 | 0 | 0 | 0 | 0 |
| 2.59406 | 0 | 0 | 0 | 0 | 0 | 0 |
| 3.63168 | 0 | 13.98674 | 0 | 11.10828 | 2.923788 | 16.20142 |
| 4.66931 | 0 | 0.386744 | 0.000303 | 0.612547 | 0.105909 | 0.828651 |
| 5.70693 | 0.000152 | 0.025763 | 0.000152 | 0.086987 | 0.000909 | 0.133966 |
| 6.74455 | 0.000455 | 0.189735 | 0.000758 | 0.908667 | 0.001212 | 0.881691 |
| 7.78218 | 0 | 0.537683 | 0.01 | 3.150024 | 0.026818 | 2.473525 |
| 8.8198 | 0 | 0.290967 | 0.050152 | 2.916947 | 0.217424 | 1.679427 |
| 9.85743 | 0.001061 | 0.129723 | 0.673636 | 1.855826 | 1.071061 | 0.86684 |
| 10.89505 | 0.001667 | 0.119721 | 0.542727 | 1.17478 | 0.402273 | 0.774094 |
| 11.93267 | 0.005758 | 0.367346 | 0.290909 | 1.115375 | 0.163182 | 1.124467 |
| 12.9703 | 0.018636 | 0.691957 | 0.223182 | 1.37573 | 0.309242 | 1.191147 |
| 14.00792 | 0.052121 | 0.772882 | 0.467576 | 0.963829 | 0.645758 | 1.230549 |
| 15.04554 | 0.127424 | 0.794098 | 0.572424 | 0.831075 | 1.03197 | 1.051725 |
| 16.08317 | 0.282424 | 0.85805 | 0.655303 | 0.881691 | 1.102727 | 0.978377 |
| 17.12079 | 0.460758 | 1.028994 | 0.600152 | 0.772579 | 0.924697 | 1.025357 |
| 18.15842 | 0.610152 | 1.103251 | 0.515758 | 0.645584 | 0.834242 | 1.142653 |
| 19.19604 | 0.85 | 0.909273 | 0.597424 | 0.603757 | 0.730303 | 1.179933 |
| 20.23366 | 0.918333 | 0.835015 | 0.694697 | 0.672862 | 0.875303 | 1.001109 |

**DFOB^–^ ρ/ρ_bulk_**

| **r(Å)** | **1^st^ charge** | **1^st^ discharge** | **2^nd^ charge** | **2^nd^ discharge** | **5^th^ charge** | **5^th^ discharge** |
| --- | --- | --- | --- | --- | --- | --- |
| 0.51881 | 0 | 0 | 0 | 0 | 0 | 0 |
| 1.55644 | 0 | 0 | 0 | 0 | 0 | 0 |
| 2.59406 | 0 | 0 | 0 | 0 | 0 | 0 |
| 3.63168 | 0.000152 | 9.324592 | 0.002879 | 11.44108 | 1.247121 | 11.08494 |
| 4.66931 | 0.000758 | 0.534652 | 0.077727 | 0.756212 | 1.139848 | 0.875023 |
| 5.70693 | 0.083939 | 1.603048 | 0.689848 | 1.598502 | 1.174697 | 1.678214 |
| 6.74455 | 0.984545 | 0.670437 | 2.227727 | 0.432511 | 2.377576 | 0.538289 |
| 7.78218 | 0.412424 | 1.254796 | 0.03697 | 1.000806 | 0.122273 | 1.680033 |
| 8.8198 | 0.030303 | 1.46302 | 0.006364 | 1.639419 | 0.038636 | 2.512927 |
| 9.85743 | 0.030606 | 1.015658 | 0.170455 | 0.913819 | 0.702879 | 1.520304 |
| 10.89505 | 0.251061 | 0.768335 | 1.088333 | 0.548595 | 0.62303 | 1.038389 |
| 11.93267 | 0.441818 | 1.064455 | 2.209394 | 0.54617 | 0.566818 | 1.026872 |
| 12.9703 | 0.432424 | 1.495451 | 1.128333 | 0.737723 | 0.565606 | 1.072336 |
| 14.00792 | 0.360152 | 1.320567 | 0.384242 | 0.756212 | 0.555455 | 1.192663 |
| 15.04554 | 0.515758 | 1.203271 | 0.546818 | 0.672256 | 0.584848 | 1.001109 |
| 16.08317 | 0.718939 | 1.233883 | 0.736818 | 0.620124 | 0.578333 | 0.932308 |
| 17.12079 | 0.842121 | 1.265708 | 0.632121 | 0.659526 | 0.597576 | 0.994744 |
| 18.15842 | 0.823939 | 1.420284 | 0.606667 | 0.771973 | 0.68 | 0.997169 |
| 19.19604 | 1.087727 | 1.457867 | 0.692424 | 0.860475 | 0.800455 | 0.970194 |
| 20.23366 | 1.472121 | 1.523638 | 0.823636 | 0.940188 | 0.908636 | 1.099311 |

**DEC ρ/ρ_bulk_**

| **r(Å)** | **1^st^ charge** | **1^st^ discharge** | **2^nd^ charge** | **2^nd^ discharge** | **5^th^ charge** | **5^th^ discharge** |
| --- | --- | --- | --- | --- | --- | --- |
| 0.51881 | 0 | 0 | 0 | 0 | 0 | 0 |
| 1.55644 | 0 | 0 | 0 | 0 | 0 | 0 |
| 2.59406 | 0 | 0 | 0 | 0 | 0 | 0 |
| 3.63168 | 1.6 | 1.4 | 0.4 | 0.6 | 0.2 | 0.2 |
| 4.66931 | 1 | 0.2 | 0.2 | 0.4 | 0 | 0.6 |
| 5.70693 | 0.8 | 0.8 | 0.2 | 0.2 | 0 | 0.4 |
| 6.74455 | 1.4 | 1.2 | 1 | 1.2 | 1.2 | 1 |
| 7.78218 | 0.8 | 1 | 0.6 | 0.6 | 1 | 1.2 |
| 8.8198 | 0 | 0.8 | 0.8 | 1 | 0.6 | 0.6 |
| 9.85743 | 1.8 | 1.8 | 2.2 | 1.4 | 0.8 | 1.6 |
| 10.89505 | 1.2 | 1 | 0.4 | 0.8 | 1.2 | 1.6 |
| 11.93267 | 0.6 | 1.4 | 0.8 | 1.2 | 1.8 | 0.8 |
| 12.9703 | 1.4 | 0.8 | 1.4 | 0.6 | 1.4 | 1 |
| 14.00792 | 1.2 | 1.8 | 1.2 | 1.4 | 1 | 0.4 |
| 15.04554 | 1.2 | 1.2 | 1.2 | 0.8 | 1 | 1.8 |
| 16.08317 | 1 | 1 | 1.2 | 1 | 1.6 | 1.4 |
| 17.12079 | 1 | 0.8 | 0.4 | 0.6 | 1.4 | 0.8 |
| 18.15842 | 0.8 | 0.8 | 1.8 | 0.8 | 1.2 | 1.8 |
| 19.19604 | 1.4 | 0.6 | 0.6 | 1.4 | 1.4 | 0.2 |
| 20.23366 | 1 | 1.4 | 1.2 | 1 | 0.8 | 1 |

**FEC ρ/ρ_bulk_**

| **r(Å)** | **1^st^ charge** | **1^st^ discharge** | **2^nd^ charge** | **2^nd^ discharge** | **5^th^ charge** | **5^th^ discharge** |
| --- | --- | --- | --- | --- | --- | --- |
| 0.51881 | 0 | 0 | 0 | 0 | 0 | 0 |
| 1.55644 | 0 | 0 | 0 | 0 | 0 | 0 |
| 2.59406 | 0 | 0 | 0 | 0 | 0 | 0 |
| 3.63168 | 2.8 | 4.2 | 4.8 | 4.8 | 4 | 5.2 |
| 4.66931 | 3.2 | 3.2 | 4 | 4 | 5.2 | 3.6 |
| 5.70693 | 0.4 | 0.8 | 0.4 | 0.6 | 0.2 | 0.8 |
| 6.74455 | 0.2 | 0.2 | 0.4 | 0.4 | 0.4 | 0.2 |
| 7.78218 | 1.4 | 1 | 1.2 | 3.2 | 1.8 | 2.2 |
| 8.8198 | 1.8 | 1.2 | 1.8 | 0.8 | 1.8 | 0.8 |
| 9.85743 | 1 | 0.2 | 1.2 | 0.8 | 0.8 | 0.4 |
| 10.89505 | 1 | 1 | 0.8 | 1.6 | 0.4 | 0.2 |
| 11.93267 | 0.2 | 1 | 0.4 | 1.2 | 0.6 | 1.6 |
| 12.9703 | 1 | 1 | 2 | 1 | 0.4 | 1.4 |
| 14.00792 | 0.8 | 0.6 | 1 | 1 | 0.8 | 0.8 |
| 15.04554 | 1.6 | 1.2 | 0.6 | 0.8 | 0.6 | 0.8 |
| 16.08317 | 0.8 | 1.4 | 1.2 | 1.4 | 0.8 | 0.4 |
| 17.12079 | 0.8 | 1.2 | 0.8 | 0.8 | 0.6 | 1 |
| 18.15842 | 1.2 | 1.4 | 1.4 | 1.8 | 0.8 | 0.4 |
| 19.19604 | 1.2 | 1 | 1 | 0.8 | 0.6 | 0.4 |
| 20.23366 | 0.8 | 1 | 1.4 | 0.2 | 0.8 | 0.8 |

**Table S4.** Averaged number density values of Li^+^, TFSI^–^, DOL, and DME species within the EDL during the 1^st^, 2^nd^, and 5^th^ charge–discharge cycles in EL. 1.

**Li^+^ ρ/ρ_bulk_**

| **r(Å)** | **1^st^ charge** | **1^st^ discharge** | **2^nd^ charge** | **2^nd^ discharge** | **5^th^ charge** | **5^th^ discharge** |
| --- | --- | --- | --- | --- | --- | --- |
| 0.51881 | 0 | 0 | 0 | 0 | 0 | 0 |
| 1.55644 | 1.652364 | 0 | 2.095182 | 0 | 1.848364 | 0 |
| 2.59406 | 12.14682 | 0 | 12.33464 | 0 | 14.17945 | 0 |
| 3.63168 | 0.007364 | 0 | 0.018182 | 0 | 0.008364 | 0 |
| 4.66931 | 2.178364 | 0 | 2.089091 | 0 | 1.213182 | 0.000364 |
| 5.70693 | 3.464091 | 0.000909 | 5.063727 | 0.000182 | 2.574 | 0.000364 |
| 6.74455 | 0.118727 | 0.001637 | 0.281636 | 0.001637 | 0.123636 | 0.003819 |
| 7.78218 | 0.021455 | 0.002728 | 0.035727 | 0.002 | 0.061364 | 0.02546 |
| 8.8198 | 0.947727 | 0.061103 | 0.821364 | 0.078925 | 0.755364 | 0.090382 |
| 9.85743 | 2.599455 | 1.185874 | 2.514909 | 1.225882 | 2.234636 | 0.926913 |
| 10.89505 | 0.581636 | 2.842205 | 0.720909 | 2.393388 | 0.605727 | 2.016403 |
| 11.93267 | 0.263727 | 1.49248 | 0.321 | 1.47775 | 0.270091 | 1.222063 |
| 12.9703 | 0.484273 | 0.946007 | 0.667636 | 0.923639 | 0.562909 | 0.729782 |
| 14.00792 | 0.823091 | 1.498482 | 1.240545 | 1.510484 | 1.024 | 1.099129 |
| 15.04554 | 0.660818 | 1.509938 | 1.071545 | 1.546309 | 0.855455 | 1.19242 |
| 16.08317 | 0.609818 | 0.894724 | 0.946818 | 0.836895 | 0.716 | 0.778701 |
| 17.12079 | 0.734727 | 0.783066 | 1.114727 | 0.673226 | 0.858 | 0.661951 |
| 18.15842 | 0.907727 | 0.934369 | 1.220455 | 0.745786 | 1.047636 | 0.818527 |
| 19.19604 | 0.928364 | 1.0453 | 1.032818 | 0.788703 | 1.068818 | 0.827256 |
| 20.23366 | 0.899273 | 1.011657 | 0.881818 | 0.813981 | 0.978545 | 0.814163 |

**TFSI^–^ ρ/ρ_bulk_**

| **r(Å)** | **1^st^ charge** | **1^st^ discharge** | **2^nd^ charge** | **2^nd^ discharge** | **5^th^ charge** | **5^th^ discharge** |
| --- | --- | --- | --- | --- | --- | --- |
| 0.51881 | 0 | 0 | 0 | 0 | 0 | 0 |
| 1.55644 | 0 | 0 | 0 | 0 | 0 | 0 |
| 2.59406 | 0 | 0 | 0 | 0 | 0 | 0 |
| 3.63168 | 0 | 3.463238 | 0 | 3.294659 | 0 | 3.389769 |
| 4.66931 | 9.09E-05 | 11.91766 | 0.007182 | 11.05548 | 0 | 11.51139 |
| 5.70693 | 0.000273 | 0.038735 | 0.007091 | 0.049101 | 0.000636 | 0.064377 |
| 6.74455 | 0.000455 | 0.084744 | 0.014364 | 0.170216 | 0.002455 | 0.133481 |
| 7.78218 | 0.033818 | 1.219335 | 0.221909 | 1.873829 | 0.080818 | 1.342268 |
| 8.8198 | 0.263545 | 1.918747 | 1.091273 | 2.457582 | 0.433182 | 1.704705 |
| 9.85743 | 0.648364 | 1.224063 | 1.630182 | 1.353362 | 0.681909 | 0.825983 |
| 10.89505 | 0.837273 | 1.030024 | 1.546636 | 1.014385 | 0.734636 | 0.613032 |
| 11.93267 | 0.771909 | 0.88745 | 1.371273 | 0.814527 | 0.706636 | 0.586481 |
| 12.9703 | 0.873818 | 0.820528 | 1.263 | 0.634491 | 0.955455 | 0.696867 |
| 14.00792 | 0.956364 | 0.976377 | 1.110182 | 0.74233 | 1.097455 | 0.95219 |
| 15.04554 | 0.960545 | 1.138955 | 0.882 | 0.888723 | 0.992818 | 0.954373 |
| 16.08317 | 0.946909 | 1.166597 | 0.876455 | 0.885268 | 1.008909 | 0.907818 |
| 17.12079 | 0.896636 | 1.323719 | 0.903364 | 1.003655 | 1.070909 | 0.980378 |
| 18.15842 | 0.848 | 1.246613 | 0.841 | 1.089491 | 0.969909 | 1.020568 |
| 19.19604 | 0.763 | 1.169325 | 0.855091 | 1.020568 | 0.835182 | 0.960919 |
| 20.23366 | 0.712545 | 0.95219 | 0.962091 | 0.95801 | 0.839273 | 0.893633 |

**DOL ρ/ρ_bulk_**

| **r(Å)** | **1^st^ charge** | **1^st^ discharge** | **2^nd^ charge** | **2^nd^ discharge** | **5^th^ charge** | **5^th^ discharge** |
| --- | --- | --- | --- | --- | --- | --- |
| 0.51881 | 0 | 0 | 0 | 0 | 0 | 0 |
| 1.55644 | 0 | 0 | 0 | 0 | 0 | 0 |
| 2.59406 | 0 | 0 | 0 | 0 | 0 | 0 |
| 3.63168 | 2.702703 | 4.633205 | 3.667954 | 2.509653 | 3.088803 | 5.019305 |
| 4.66931 | 5.212355 | 2.895753 | 1.544402 | 2.702703 | 2.316602 | 2.509653 |
| 5.70693 | 0 | 0 | 0 | 0.3861 | 0.19305 | 0 |
| 6.74455 | 0 | 0.19305 | 0.19305 | 0 | 0 | 0.3861 |
| 7.78218 | 2.123552 | 1.737452 | 0.3861 | 1.351351 | 1.544402 | 2.509653 |
| 8.8198 | 2.509653 | 1.351351 | 1.158301 | 1.351351 | 1.544402 | 1.351351 |
| 9.85743 | 0.3861 | 0.3861 | 0.965251 | 0.965251 | 0.965251 | 1.544402 |
| 10.89505 | 0.965251 | 0.579151 | 0.19305 | 0.965251 | 0.772201 | 0.19305 |
| 11.93267 | 1.544402 | 0.965251 | 1.351351 | 0.3861 | 1.158301 | 1.351351 |
| 12.9703 | 1.737452 | 1.351351 | 1.158301 | 0.772201 | 2.123552 | 0.965251 |
| 14.00792 | 1.158301 | 1.737452 | 0.3861 | 0.772201 | 0.772201 | 0.579151 |
| 15.04554 | 1.544402 | 0.579151 | 0.772201 | 0.3861 | 0.19305 | 1.544402 |
| 16.08317 | 0.19305 | 1.351351 | 1.158301 | 0.579151 | 0.772201 | 2.123552 |
| 17.12079 | 1.158301 | 0.772201 | 0.772201 | 1.158301 | 0.772201 | 0.772201 |
| 18.15842 | 0.965251 | 0.772201 | 0.965251 | 1.158301 | 2.123552 | 0.772201 |
| 19.19604 | 1.351351 | 1.158301 | 0.3861 | 0.772201 | 1.351351 | 0.3861 |
| 20.23366 | 0.965251 | 1.158301 | 1.544402 | 0.772201 | 0.772201 | 0.579151 |

**DME ρ/ρ_bulk_**

| **r(Å)** | **1^st^ charge** | **1^st^ discharge** | **2^nd^ charge** | **2^nd^ discharge** | **5^th^ charge** | **5^th^ discharge** |
| --- | --- | --- | --- | --- | --- | --- |
| 0.51881 | 0 | 0 | 0 | 0 | 0 | 0 |
| 1.55644 | 0 | 0 | 0 | 0 | 0 | 0 |
| 2.59406 | 0 | 0 | 0 | 0 | 0 | 0 |
| 3.63168 | 0.965251 | 0.579151 | 0.772201 | 1.544402 | 1.737452 | 0.579151 |
| 4.66931 | 9.65E-01 | 1.158301 | 1.737452 | 1.930502 | 0.3861 | 1.158301 |
| 5.70693 | 1.158301 | 1.351351 | 0.965251 | 1.158301 | 0.772201 | 1.158301 |
| 6.74455 | 0.3861 | 0.772201 | 0.19305 | 0.772201 | 0 | 1.158301 |
| 7.78218 | 1.158301 | 0.965251 | 1.351351 | 0.772201 | 1.351351 | 0.579151 |
| 8.8198 | 0.579151 | 0.965251 | 1.544402 | 0.772201 | 1.544402 | 0.19305 |
| 9.85743 | 1.351351 | 1.158301 | 1.351351 | 0.772201 | 0.579151 | 0.19305 |
| 10.89505 | 0.772201 | 0.19305 | 0.772201 | 0.579151 | 0.579151 | 0.772201 |
| 11.93267 | 0.3861 | 1.544402 | 1.544402 | 0.19305 | 1.737452 | 1.158301 |
| 12.9703 | 0.772201 | 0.965251 | 1.351351 | 1.544402 | 1.351351 | 1.351351 |
| 14.00792 | 1.351351 | 0.965251 | 1.158301 | 1.158301 | 1.351351 | 1.351351 |
| 15.04554 | 1.544402 | 0.965251 | 0.579151 | 1.351351 | 1.544402 | 0.965251 |
| 16.08317 | 0.3861 | 0.3861 | 1.351351 | 0.965251 | 0.965251 | 0.579151 |
| 17.12079 | 1.351351 | 2.123552 | 1.544402 | 1.158301 | 1.158301 | 0.772201 |
| 18.15842 | 1.544402 | 0.579151 | 1.158301 | 0.965251 | 1.158301 | 1.544402 |
| 19.19604 | 0.3861 | 0.579151 | 1.158301 | 1.737452 | 0.965251 | 1.544402 |
| 20.23366 | 0.965251 | 0.772201 | 1.544402 | 0.772201 | 1.351351 | 0.772201 |

**Table S5.** Averaged number density values of Li^+^, BF_4_^–^, DFOB^–^, DEC, and FEC species within the EDL during the 1^st^, 2^nd^, and 5^th^ charge–rest cycles in EL. 4.

**Li^+^ ρ/ρ_bulk_**

| **r(Å)** | **1^st^ charge** | **1^st^ rest** | **2^nd^ charge** | **2^nd^ rest** | **5^th^ charge** | **5^th^ rest** |
| --- | --- | --- | --- | --- | --- | --- |
| 0.51881 | 0 | 0 | 0 | 0 | 0 | 0 |
| 1.55644 | 2.886742 | 0 | 2.181515 | 0 | 2.209773 | 0 |
| 2.59406 | 5.629924 | 0.001818 | 6.366894 | 0.013482 | 5.342879 | 0.000454 |
| 3.63168 | 0.001439 | 0.801355 | 0.017652 | 1.136439 | 0.001061 | 0.389468 |
| 4.66931 | 0.179697 | 1.362758 | 0.031136 | 1.281713 | 0.060379 | 0.605939 |
| 5.70693 | 0.490455 | 0.289942 | 0.147273 | 0.369926 | 0.599621 | 0.244497 |
| 6.74455 | 0.729621 | 0.797416 | 1.020455 | 0.520956 | 2.134242 | 0.473845 |
| 7.78218 | 4.658939 | 1.816152 | 4.209621 | 1.004799 | 4.585909 | 1.240812 |
| 8.8198 | 2.123106 | 1.968697 | 1.905076 | 1.613768 | 1.656061 | 2.43118 |
| 9.85743 | 0.274318 | 0.946932 | 0.325909 | 1.151285 | 0.121667 | 1.006768 |
| 10.89505 | 0.578258 | 1.591197 | 0.597727 | 1.812213 | 0.524773 | 1.153557 |
| 11.93267 | 0.894545 | 1.811304 | 0.98447 | 2.011416 | 1.015606 | 1.374119 |
| 12.9703 | 0.781591 | 1.212333 | 0.722576 | 1.606345 | 0.818333 | 1.61195 |
| 14.00792 | 0.614242 | 0.826047 | 0.681061 | 1.431683 | 0.754697 | 1.412596 |
| 15.04554 | 0.607803 | 0.810595 | 0.81053 | 1.418353 | 0.914773 | 1.063272 |
| 16.08317 | 0.780606 | 0.900274 | 0.842652 | 1.111141 | 0.949773 | 1.107203 |
| 17.12079 | 0.841591 | 0.775148 | 0.977348 | 0.923906 | 0.799621 | 0.880581 |
| 18.15842 | 0.895606 | 0.753334 | 0.901742 | 0.798931 | 0.737879 | 0.989045 |
| 19.19604 | 0.843939 | 0.855132 | 0.810455 | 0.600334 | 0.829091 | 0.982531 |
| 20.23366 | 0.893333 | 0.893003 | 0.789773 | 0.635327 | 0.833485 | 1.019796 |

**BF_4_^–^ ρ/ρ_bulk_**

| **r(Å)** | **1^st^ charge** | **1^st^ rest** | **2^nd^ charge** | **2^nd^ rest** | **5^th^ charge** | **5^th^ rest** |
| --- | --- | --- | --- | --- | --- | --- |
| 0.51881 | 0 | 0 | 0 | 0 | 0 | 0 |
| 1.55644 | 0 | 0 | 0 | 0 | 0 | 0 |
| 2.59406 | 0 | 0 | 0 | 0 | 0 | 0 |
| 3.63168 | 0 | 0.86619 | 1.337576 | 0.348112 | 1.329091 | 0.030903 |
| 4.66931 | 0 | 0.671684 | 0.177576 | 0.337811 | 0.184697 | 0.055746 |
| 5.70693 | 0 | 0.220562 | 0.000152 | 0.192689 | 0.001212 | 0.073016 |
| 6.74455 | 0 | 0.36629 | 0.000455 | 0.370532 | 0.000152 | 0.783177 |
| 7.78218 | 0 | 1.436682 | 0.008636 | 0.598971 | 0 | 2.916689 |
| 8.8198 | 0.000152 | 0.677137 | 0.118485 | 0.415069 | 0.001667 | 1.448195 |
| 9.85743 | 0.002424 | 0.324481 | 0.960303 | 0.54171 | 0.017121 | 0.897396 |
| 10.89505 | 0.007879 | 1.007071 | 0.415303 | 1.597256 | 0.040909 | 2.482534 |
| 11.93267 | 0.002879 | 0.895578 | 0.015455 | 2.381948 | 0.021061 | 2.779141 |
| 12.9703 | 0.019545 | 0.737731 | 0.034091 | 2.007174 | 0.085 | 1.424261 |
| 14.00792 | 0.081515 | 0.829228 | 0.081364 | 1.584229 | 0.289091 | 1.068574 |
| 15.04554 | 0.191061 | 0.943448 | 0.18803 | 1.730563 | 0.60803 | 0.871644 |
| 16.08317 | 0.414394 | 1.004648 | 0.325152 | 1.617252 | 0.747273 | 0.690468 |
| 17.12079 | 0.605152 | 0.814383 | 0.475909 | 1.143711 | 0.86 | 0.734702 |
| 18.15842 | 0.633182 | 0.661989 | 0.536515 | 0.868917 | 1.017424 | 0.690468 |
| 19.19604 | 0.585606 | 0.636236 | 0.626364 | 0.700466 | 1.14803 | 0.69471 |
| 20.23366 | 0.570152 | 0.638963 | 0.637121 | 0.758939 | 1.188485 | 0.789539 |

**DFOB^–^ ρ/ρ_bulk_**

| **r(Å)** | **1^st^ charge** | **1^st^ rest** | **2^nd^ charge** | **2^nd^ rest** | **5^th^ charge** | **5^th^ rest** |
| --- | --- | --- | --- | --- | --- | --- |
| 0.51881 | 0 | 0 | 0 | 0 | 0 | 0 |
| 1.55644 | 0 | 0 | 0 | 0 | 0 | 0 |
| 2.59406 | 0 | 0 | 0 | 0 | 0 | 0 |
| 3.63168 | 0.001364 | 1.093418 | 0.001212 | 1.309435 | 0 | 0.600486 |
| 4.66931 | 0.086515 | 0.68865 | 0.032879 | 0.790448 | 0.006818 | 0.571098 |
| 5.70693 | 1.543939 | 0.182085 | 0.288636 | 0.070895 | 0.911515 | 0.146637 |
| 6.74455 | 1.392879 | 1.468191 | 1.051364 | 0.844074 | 0.595606 | 0.350839 |
| 7.78218 | 0.017727 | 5.011725 | 0.085152 | 3.226627 | 0.082121 | 1.334279 |
| 8.8198 | 0.091061 | 2.497682 | 0.009848 | 1.536359 | 0.230758 | 0.768634 |
| 9.85743 | 0.213485 | 1.425775 | 0.054242 | 1.210667 | 0.428939 | 0.560797 |
| 10.89505 | 0.519848 | 1.426987 | 0.185758 | 2.075948 | 0.703788 | 0.944963 |
| 11.93267 | 1.137576 | 1.598771 | 0.241061 | 2.782171 | 0.771515 | 1.381542 |
| 12.9703 | 0.839697 | 1.160374 | 0.221061 | 2.070495 | 0.777879 | 1.406385 |
| 14.00792 | 0.608788 | 0.866796 | 0.376818 | 1.295196 | 0.420303 | 1.222483 |
| 15.04554 | 0.755 | 1.042822 | 0.788333 | 0.828622 | 0.501061 | 1.312768 |
| 16.08317 | 0.881061 | 0.819533 | 1.308636 | 0.797113 | 0.53197 | 1.387601 |
| 17.12079 | 0.938333 | 0.708343 | 1.28303 | 0.756515 | 0.522273 | 1.21097 |
| 18.15842 | 1.011061 | 0.684712 | 1.130303 | 0.546254 | 0.497424 | 1.098871 |
| 19.19604 | 1.255303 | 0.83559 | 1.223485 | 0.667442 | 0.494394 | 1.090388 |
| 20.23366 | 1.236515 | 0.931026 | 1.283182 | 0.656535 | 0.646061 | 1.121291 |

**DEC ρ/ρ_bulk_**

| **r(Å)** | **1^st^ charge** | **1^st^ rest** | **2^nd^ charge** | **2^nd^ rest** | **5^th^ charge** | **5^th^ rest** |
| --- | --- | --- | --- | --- | --- | --- |
| 0.51881 | 0 | 0 | 0 | 0 | 0 | 0 |
| 1.55644 | 0 | 0 | 0 | 0 | 0 | 0 |
| 2.59406 | 0 | 0 | 0 | 0 | 0 | 0 |
| 3.63168 | 1.42768 | 1.803199 | 1.10666 | 1.297141 | 1.2268 | 1.852949 |
| 4.66931 | 2.2618 | 0.287263 | 1.84666 | 0.146651 | 2.65836 | 0.329574 |
| 5.70693 | 0.19588 | 0.594641 | 0.23962 | 0.517457 | 0.26682 | 0.647111 |
| 6.74455 | 0.3189 | 1.020556 | 0.34892 | 0.940532 | 0.2946 | 1.132494 |
| 7.78218 | 0.60108 | 1.009518 | 0.47656 | 1.096381 | 0.71112 | 1.216157 |
| 8.8198 | 1.18366 | 0.593081 | 0.91522 | 0.705419 | 1.16256 | 0.675305 |
| 9.85743 | 2.55766 | 0.942931 | 2.28184 | 0.991962 | 2.53678 | 1.009238 |
| 10.89505 | 1.47666 | 1.135533 | 1.45078 | 1.091742 | 1.46476 | 1.173645 |
| 11.93267 | 0.80228 | 1.112418 | 0.73514 | 0.946731 | 0.80738 | 1.095861 |
| 12.9703 | 0.92274 | 0.910058 | 0.80544 | 0.911818 | 0.99066 | 0.943211 |
| 14.00792 | 1.17616 | 0.978444 | 1.08354 | 0.997441 | 1.25926 | 1.083263 |
| 15.04554 | 1.26088 | 0.970086 | 1.19824 | 1.04887 | 1.36586 | 1.183163 |
| 16.08317 | 1.15422 | 0.938692 | 1.13852 | 1.010878 | 1.29794 | 1.244511 |
| 17.12079 | 0.9774 | 0.94789 | 0.97708 | 0.972046 | 1.17182 | 1.14905 |
| 18.15842 | 1.0173 | 0.909098 | 0.95302 | 0.983603 | 1.0779 | 1.057748 |
| 19.19604 | 1.06872 | 0.963127 | 1.04108 | 0.973045 | 1.11512 | 1.082024 |
| 20.23366 | 1.09802 | 0.996841 | 1.0716 | 0.929374 | 1.1084 | 1.124695 |

**FEC ρ/ρ_bulk_**

| **r(Å)** | **1^st^ charge** | **1^st^ rest** | **2^nd^ charge** | **2^nd^ rest** | **5^th^ charge** | **5^th^ rest** |
| --- | --- | --- | --- | --- | --- | --- |
| 0.51881 | 0 | 0 | 0 | 0 | 0 | 0 |
| 1.55644 | 0 | 0 | 0 | 0 | 0 | 0 |
| 2.59406 | 0 | 0.00004 | 0 | 0.00012 | 0 | 0.00016 |
| 3.63168 | 0.50402 | 4.232833 | 0.70538 | 5.180564 | 0.2208 | 4.289582 |
| 4.66931 | 7.77424 | 2.605119 | 8.70894 | 2.786923 | 7.99804 | 2.563007 |
| 5.70693 | 0.02384 | 0.411398 | 0.0362 | 0.416837 | 0.01924 | 0.381804 |
| 6.74455 | 0.10018 | 0.535853 | 0.14096 | 0.694021 | 0.08576 | 0.573485 |
| 7.78218 | 1.2703 | 1.807718 | 1.50416 | 2.237632 | 1.2095 | 1.939572 |
| 8.8198 | 1.74114 | 1.386483 | 1.9621 | 1.374725 | 1.55842 | 1.303859 |
| 9.85743 | 0.67956 | 0.793361 | 0.9832 | 0.721976 | 0.63004 | 0.65199 |
| 10.89505 | 0.8334 | 0.931414 | 1.04616 | 0.820956 | 0.74652 | 0.80168 |
| 11.93267 | 0.83644 | 1.322416 | 1.00416 | 1.116497 | 0.79826 | 1.145531 |
| 12.9703 | 0.83486 | 1.184563 | 1.03004 | 0.967007 | 0.83486 | 0.94829 |
| 14.00792 | 0.87828 | 0.927495 | 1.00576 | 0.812238 | 0.78462 | 0.718416 |
| 15.04554 | 0.92294 | 1.054949 | 1.03804 | 0.937373 | 0.80818 | 0.759728 |
| 16.08317 | 0.9656 | 1.286903 | 1.06402 | 1.174725 | 0.83036 | 0.892781 |
| 17.12079 | 0.98486 | 1.288342 | 1.02872 | 1.138132 | 0.81866 | 0.889662 |
| 18.15842 | 0.94298 | 1.204719 | 0.96588 | 1.104139 | 0.79722 | 0.820836 |
| 19.19604 | 0.96318 | 1.180164 | 0.96972 | 1.168926 | 0.86976 | 0.855869 |
| 20.23366 | 1.02204 | 1.188482 | 1.00572 | 1.243311 | 0.93796 | 0.928774 |

**Table S6.** Distributions of Li^+^ solvation states in the EDL of EL. 4 during the charge and discharge steps of the 1^st^ and 5^th^ cycles under the reflex charging protocol, corresponding to Figure 3a–d.

| **Solvation structure** | **1^st^ charge** | **1^st^ discharge** | **5^th^ charge** | **5^th^ discharge** |
| --- | --- | --- | --- | --- |
| LiFEC | – | – | 0.05516 | – |
| Li_2_FEC | – | – | 0.05864 | – |
| LiDEC | 0.04397 | – | 0.05828 | – |
| LiDECFEC | 0.0628 | – | 0.09694 | – |
| Li_2_DEC | 0.26852 | 0.02331 | 0.03285 | – |
| Li_2_DECFEC | 0.11492 | – | – | – |
| Li_2_(DEC)_2_FEC | 0.08615 | – | 0.04848 | – |
| Li_2_(DEC)_3_FEC | 0.03015 | – | 0.05206 | – |
| Li_3_DEC | 0.06033 | 0.03694 | 0.04229 | – |
| Li_3_DECFEC | 0.1137 | – | 0.0486 | – |
| Li_3_(DEC)_2_FEC | 0.05535 | – | – | – |
| LiBF_4_DFOBDEC | – | – | – | 0.01997 |
| LiBF_4_(DFOB)_2_DEC | – | – | – | 0.04787 |
| LiDFOBDEC | – | – | 0.04108 | – |
| Li(DFOB)_2_DEC | – | – | – | 0.02196 |
| Li(DFOB)_2_DECFEC | – | – | 0.05206 | – |
| Li(DFOB)_3_DEC | – | 0.03082 | 0.04122 | 0.02596 |
| LiBF_4_ | – | – | 0.02536 | – |
| LiBF_4_FEC | – | – | 0.03803 | – |
| LiBF_4_DEC | – | – | 0.09286 | – |
| LiBF_4_DECFEC | – | – | 0.03447 | – |
| Li(BF_4_)_2_DEC | – | 0.02481 | 0.02992 | 0.03232 |
| Li(BF_4_)_2_DECFEC | – | – | 0.04752 | – |
| Li(BF_4_)_3_DEC | – | 0.03009 | 0.02773 | 0.02543 |
| Li_2_BF_4_DEC | – | 0.02829 | – | 0.03598 |
| Li_2_(BF_4_)_2_DEC | – | – | – | 0.02698 |
| Li_2_BF_4_DFOBDEC | – | – | – | 0.02051 |
| Li_3_BF_4_DEC | – | – | – | 0.02383 |
| Free BF_4_ | 0.00287 | 0.2821 | – | 0.20391 |
| Free DFOB | 0.01053 | 0.38962 | – | 0.26247 |
| **Total** | **0.84929** | **0.84598** | **0.92355** | **0.74719** |

**Table S7.** Distributions of Li^+^ solvation states in the EDL of EL. 4 under CC charging, compared under conditions matched to the total charging duration or total charge of 5 charge–discharge cycles under reflex charging.

| **Solvation structure** | **CC (duration-matched to reflex)** | **CC (charge-matched to reflex)** |
| --- | --- | --- |
| LiDEC | 0.10393 | 0.10721 |
| LiDECFEC | 0.10683 | 0.11194 |
| Li(DEC)_2_FEC | 0.02857 | 0.03655 |
| Li_2_DEC | 0.17224 | 0.16712 |
| Li_2_DECFEC | 0.07681 | 0.06864 |
| Li_2_(DEC)_2_FEC | 0.07212 | 0.08064 |
| Li_2_(DEC)_3_FEC | 0.02335 | 0.02715 |
| Li_3_DEC | 0.05967 | 0.04481 |
| Li_3_DECFEC | 0.0847 | 0.08424 |
| Li_3_(DEC)_2_FEC | 0.03021 | 0.03167 |
| LiDFOBDEC | 0.09345 | 0.09428 |
| LiDFOBDECFEC | 0.03573 | 0.02625 |
| Li(BF_4_)_2_DEC | **–** | 0.02036 |
| Li(BF_4_)_3_DEC | **–** | 0.02197 |
| Free BF_4_ | 0 | 0 |
| Free DFOB | 0 | 0 |
| **Total** | **0.88761** | **0.92283** |

**Table S8.** Comparison of full cell performance metrics for reflex charging and recent anion-enrichment related strategies in Li metal batteries

| **Type of cell** | **Strategy category** | **Areal capacity [mAh cm^–2^]** | **N/P ratio** | **Cycle life** | **Capacity retention [%]** | **Current density [Charge/ Discharge]** | **Ref.** |
| --- | --- | --- | --- | --- | --- | --- | --- |
| LFP\|\|Li | Reflex charging protocol | 3.2 | 1.2 | 500 | 73 | 0.5 C/0.5 C | This study |
| LFP\|\|Li | BF_3_-assisted LiNO_3_ additive electrolyte | 1.58 | 3 | 300 | 90 | 0.3 C/0.6 C | [30] |
| LFP\|\|Li | Cationic surfactant additive | 1.78 | 12 | 180 | 80 | 0.5 C/0.5 C | [31] |
| LFP\|\|Li | Amphiphilic molecule additive | 2.04 | 1.7 | 500 | 80.2 | 0.5 C/0.5 C | [32] |
| LFP\|\|Li | Desolvation interfacial layer | 1.7 | 2.87 | 470 | 80 | 1 C/1 C | [33] |
| NCM811\|\|Li | Bianionic solvation electrolyte | 3.8 | 2.5 | 270 | 81 | 0.5 C/0.5 C | [34] |
| NCM811\|\|Li | Salt-philic/solvent-phobic coating | 2.5 | 4.1 | 400 | 80 | 0.2 C/0.33 C | [35] |

**4. References**

[1] A. Masias, N. Felten, R. Garcia-Mendez, J. Wolfenstine, and J. Sakamoto, “Elastic, Plastic, and Creep Mechanical Properties of Lithium Metal,” *Journal of Materials Science* **54** (2019): 2585–2600, <https://doi.org/10.1007/s10853-018-2971-3>.
[2] K. Li, J. Wang, Q. Shen, Y. Song, R. Gao, and Y. Wang, “Interfacial Design Strategy for Polymeric Lithium Metal Batteries with Superfast Charge-Transfer Kinetics,” *Advanced Energy Materials* **14** (2024): 2400956, <https://doi.org/10.1002/aenm.202400956>.
[3] Y. Kondo, T. Abe, and Y. Yamada, “Kinetics of Interfacial Ion Transfer in Lithium-Ion Batteries: Mechanism Understanding and Improvement Strategies,” *ACS Applied Materials & Interfaces* **14** (2022): 22706–22718, <https://doi.org/10.1021/acsami.1c21683>.
[4] D. Van Der Spoel, E. Lindahl, B. Hess, G. Groenhof, A. E. Mark, and H. J. C. Berendsen, “GROMACS: Fast, Flexible, and Free,” *Journal of Computational Chemistry* **26** (2005): 1701–1718, <https://doi.org/10.1002/jcc.20291>.
[5] W. L. Jorgensen, D. S. Maxwell, and J. Tirado-Rives, “Development and Testing of the OPLS All-Atom Force Field on Conformational Energetics and Properties of Organic Liquids,” *Journal of the American Chemical Society* **118** (1996): 11225–11236, <https://doi.org/10.1021/ja9621760>.
[6] L. S. Dodda, I. Cabeza de Vaca, J. Tirado-Rives, and W. L. Jorgensen, “LigParGen Web Server: An Automatic OPLS-AA Parameter Generator for Organic Ligands,” *Nucleic Acids Research* **45** (2017): W331–W336, <https://doi.org/10.1093/nar/gkx312>.
[7] J. N. Canongia Lopes, J. Deschamps, and A. A. H. Pádua, “Modeling Ionic Liquids Using a Systematic All-Atom Force Field,” *The Journal of Physical Chemistry B* **108** (2004): 2038–2047, <https://doi.org/10.1021/jp0362133>.
[8] M. Fischer, A. Heuer, and D. Diddens, “Structure and Transport Properties of Poly(ethylene oxide)-Based Cross-Linked Polymer Electrolytes—A Molecular Dynamics Simulations Study,” *Macromolecules* **55** (2022): 10229–10242, <https://doi.org/10.1021/acs.macromol.2c01795>.
[9] Y.-L. Wang, F. U. Shah, S. Glavatskih, O. N. Antzutkin, and A. Laaksonen, “Atomistic Insight into Orthoborate-Based Ionic Liquids: Force Field Development and Evaluation,” *The Journal of Physical Chemistry B* **118** (2014): 8711–8723, <https://doi.org/10.1021/jp503029d>.
[10] I. Leontyev and A. Stuchebrukhov, “Accounting for Electronic Polarization in Non-Polarizable Force Fields,” *Physical Chemistry Chemical Physics* **13** (2011): 2613–2626, <https://doi.org/10.1039/c0cp01971b>.
[11] V. Chaban, “Polarizability versus Mobility: Atomistic Force Field for Ionic Liquids,” *Physical Chemistry Chemical Physics* **13** (2011): 16055–16062, <https://doi.org/10.1039/c1cp21379b>.
[12] A. Stukowski, “Visualization and Analysis of Atomistic Simulation Data with OVITO–the Open Visualization Tool,” *Modelling and Simulation in Materials Science and Engineering* **18** (2010): 015012, <https://doi.org/10.1088/0965-0393/18/1/015012>.
[13] W. Humphrey, A. Dalke, and K. Schulten, “VMD: Visual Molecular Dynamics,” *Journal of Molecular Graphics* **14** (1996): 33–38, <https://doi.org/10.1016/0263-7855(96)00018-5>.
[14] H. J. C. Berendsen, J. P. M. Postma, W. F. van Gunsteren, A. DiNola, and J. R. Haak, “Molecular Dynamics with Coupling to an External Bath,” *The Journal of Chemical Physics* **81** (1984): 3684–3690, <https://doi.org/10.1063/1.448118>.
[15] G. Bussi, D. Donadio, and M. Parrinello, “Canonical Sampling through Velocity Rescaling,” *The Journal of Chemical Physics* **126** (2007): 014101, <https://doi.org/10.1063/1.2408420>.
[16] S. Begić, E. Jónsson, F. Chen, and M. Forsyth, “Molecular Dynamics Simulations of Pyrrolidinium and Imidazolium Ionic Liquids at Graphene Interfaces,” *Physical Chemistry Chemical Physics* **19** (2017): 30010–30020, [https://doi.org/10.1039/c7cp03389c](https://doi.org/10.1039/C7CP03389C).
[17] P. S. Crozier, R. L. Rowley, and D. Henderson, “Molecular-Dynamics Simulations of Ion Size Effects on the Fluid Structure of Aqueous Electrolyte Systems between Charged Model Electrodes,” *The Journal of Chemical Physics* **114** (2001): 7513–7517, <https://doi.org/10.1063/1.1362290>.
[18] J. Yang, Z. Bo, H. Yang, et al., “Reliability of Constant Charge Method for Molecular Dynamics Simulations on EDLCs in Nanometer and Sub-Nanometer Spaces,” *ChemElectroChem* **4** (2017): 2486–2493, <https://doi.org/10.1002/celc.201700447>.
[19] J.-P. Ryckaert, G. Ciccotti, and H. J. C. Berendsen, “Numerical Integration of the Cartesian Equations of Motion of a System with Constraints: Molecular Dynamics of n-Alkanes,” *Journal of Computational Physics* **23** (1977): 327–341, <https://doi.org/10.1016/0021-9991(77)90098-5>.
[20] B. Hess, H. Bekker, H. J. C. Berendsen, and J. G. E. M. Fraaije, “LINCS: A Linear Constraint Solver for Molecular Simulations,” *Journal of Computational Chemistry* **18** (1997): 1463–1472, [https://doi.org/10.1002/(SICI)1096-987X(199709)18:12<1463::AID-JCC4>3.0.CO;2-H](https://doi.org/10.1002/(SICI)1096-987X(199709)18:12%3C1463::AID-JCC4%3E3.0.CO;2-H).
[21] H. G. Petersen, “Accuracy and Efficiency of the Particle Mesh Ewald Method,” *The Journal of Chemical Physics* **103** (1995): 3668–3679, <https://doi.org/10.1063/1.470043>.
[22] Y.-S. Lin, G.-D. Li, S.-P. Mao, and J.-D. Chai, “Long-Range Corrected Hybrid Density Functionals with Improved Dispersion Corrections,” *Journal of Chemical Theory and Computation* **9** (2013): 263–272, <https://doi.org/10.1021/ct300715s>.
[23] F. Weigend and R. Ahlrichs, “Balanced Basis Sets of Split Valence, Triple Zeta Valence and Quadruple Zeta Valence Quality for H to Rn: Design and Assessment of Accuracy,” *Physical Chemistry Chemical Physics* **7** (2005): 3297–3305, <https://doi.org/10.1039/b508541a>.
[24] D. Rappoport and F. Furche, “Property-Optimized Gaussian Basis Sets for Molecular Response Calculations,” *The Journal of Chemical Physics* **133** (2010): 134105, <https://doi.org/10.1063/1.3484283>.
[25] F. Neese, “The ORCA Program System,” *WIREs Computational Molecular Science* **2** (2012): 73–78, <https://doi.org/10.1002/wcms.81>.
[26] F. Neese, “Software Update: The ORCA Program System—Version 5.0,” *WIREs Computational Molecular Science* **12** (2022): e1606, <https://doi.org/10.1002/wcms.1606>.
[27] V. Barone and M. Cossi, “Quantum Calculation of Molecular Energies and Energy Gradients in Solution by a Conductor Solvent Model,” *The Journal of Physical Chemistry* *A* **102** (1998): 1995–2001, <https://doi.org/10.1021/jp9716997>.
[28] A. Klamt and G. Schüürmann, “COSMO: A New Approach to Dielectric Screening in Solvents with Explicit Expressions for the Screening Energy and Its Gradient,” *Journal of the Chemical Society, Perkin Transactions* **2** (1993): 799–805, <https://doi.org/10.1039/P29930000799>.
[29] K. Kim, I. Park, S.-Y. Ha, et al., “Understanding the Thermal Instability of Fluoroethylene Carbonate in LiPF_6_-Based Electrolytes for Lithium Ion Batteries,” *Electrochimica Acta* **225** (2017): 358–368, <https://doi.org/10.1016/j.electacta.2016.12.126>.
[30] J. Zhong, Z. Wang, X. Yi, et al., “Breaking the Solubility Limit of LiNO_3_ in Carbonate Electrolyte Assisted by BF_3_ to Construct a Stable SEI Film for Dendrite-Free Lithium Metal Batteries,” *Small* **20** (2024): 2308678, <https://doi.org/10.1002/smll.202308678>.
[31] Z. Sun, J. Yang, H. Xu, et al., “Enabling an Inorganic-Rich Interface via Cationic Surfactant for High-Performance Lithium Metal Batteries,” *Nano-Micro Letters* **16** (2024): 141, <https://doi.org/10.1007/s40820-024-01364-x>.
[32] S. Liu, Y. Yan, R. Zheng, G. Tian, X. Wang, and C. Shu, “An Amphiphilic Molecule Induced Anion-Enrichment Interface for Next-Generation Lithium Metal Batteries,” *Small* **21** (2025): 2407855, <https://doi.org/10.1002/smll.202407855>.
[33] Z. Qu, K. Chen, W. Wang, Y. Dai, X. Lu, and S.-S. Lyu, “Interfacial Layers with Desolvation Function Induced Stable Deposition of Lithium Metal for Long-Cycling Lithium Metal Batteries,” *Nano Letters* **24** (2024): 8055–8062, <https://doi.org/10.1021/acs.nanolett.4c01738>.
[34] M. Sun, Y. Xie, C. Zhong, et al., “Bianionic Coordination Solvation Structure Electrolyte for High-Voltage Lithium Metal Batteries,” *Energy Storage Materials* **65** (2024): 103166, <https://doi.org/10.1016/j.ensm.2023.103166>.
[35] Z. Huang, J.-C. Lai, S.-L. Liao, et al., “A Salt-Philic, Solvent-Phobic Interfacial Coating Design for Lithium Metal Electrodes,” *Nature Energy* **8** (2023): 577–585, <https://doi.org/10.1038/s41560-023-01252-5>.
